# FTO facilitates cancer metastasis by modifying the m^6^A level of FAP to induce integrin/FAK signaling in non-small cell lung cancer

**DOI:** 10.1186/s12964-023-01343-6

**Published:** 2023-11-02

**Authors:** Lirong Gao, Anqi Wang, Yuling Chen, Xin Cai, Yue Li, Jian Zhao, Yang Zhang, Weijie Zhang, Jianjie Zhu, Yuanyuan Zeng, Zeyi Liu, Jian-an Huang

**Affiliations:** 1 Department of Pulmonary and Critical Care Medicine, the First Affiliated Hospital of Soochow University, Suzhou, 215006 China; 2 Institute of Respiratory Diseases, Soochow University, Suzhou, 215006 China; 3 Suzhou Key Laboratory for Respiratory Diseases, Suzhou, 215006 China

**Keywords:** N^6^-methyladenosine (m^6^A), Fat mass and obesity-associated protein (FTO), Fibroblast activation protein (FAP), Carcinoma, Non-Small-Cell Lung (NSCLC), Metastasis, Integrin, FAK signaling

## Abstract

**Background:**

Emerging evidence suggests the critical roles of N^6^-methyladenosine (m^6^A) RNA modification in tumorigenesis and tumor progression. However, the role of m^6^A in non-small cell lung cancer (NSCLC) is still unclear. This study aimed to explore the role of the m^6^A demethylase fat mass and obesity-associated protein (FTO) in the tumor metastasis of NSCLC.

**Methods:**

A human m^6^A epitranscriptomic microarray analysis was used to identify downstream targets of FTO. Quantitative real-time PCR (qRT‒PCR) and western blotting were employed to evaluate the expression levels of FTO and FAP in NSCLC cell lines and tissues. Gain-of-function and loss-of-function assays were conducted in vivo and in vitro to assess the effects of FTO and FAP on NSCLC metastasis. M^6^A-RNA immunoprecipitation (MeRIP), RNA immunoprecipitation (RIP), luciferase reporter assays, and RNA stability assays were used to explore the mechanism of FTO action. Co-immunoprecipitation (co-IP) assays were used to determine the mechanism of FAP in NSCLC metastasis.

**Results:**

FTO was upregulated and predicted poor prognosis in patients with NSCLC. FTO promoted cell migration and invasion in NSCLC, and the FAK inhibitor defactinib (VS6063) suppressed NSCLC metastasis induced by overexpression of FTO. Mechanistically, FTO facilitated NSCLC metastasis by modifying the m^6^A level of FAP in a YTHDF2-dependent manner. Moreover, FTO-mediated metastasis formation depended on the interactions between FAP and integrin family members, which further activated the FAK signaling.

**Conclusion:**

Our current findings provided valuable insights into the role of FTO-mediated m^6^A demethylation modification in NSCLC metastasis. FTO was identified as a contributor to NSCLC metastasis through the activation of the FAP/integrin/FAK signaling, which may be a potential therapeutic target for NSCLC.

Video Abstract

**Supplementary Information:**

The online version contains supplementary material available at 10.1186/s12964-023-01343-6.

## Abstract (plain language summary)

Emerging evidence suggests the crucial roles of N^6^-methyladenosine (m^6^A) RNA modification in tumorigenesis and progression. Nonetheless, the role of m^6^A in NSCLC remains unclear. The purpose of this study was to investigate the role of m^6^A demethylase fat mass and obesity-associated protein (FTO) in the tumor metastasis of non-small cell lung cancer (NSCLC). Results illustrated that FTO was upregulated and predicted poor prognosis in NSCLC patients. FTO promoted cell migration and invasion in NSCLC, and the FAK inhibitor defactinib (VS6063) suppressed NSCLC metastasis induced by overexpression of FTO. Mechanistically, FTO facilitated NSCLC metastasis by modifying the m^6^A level of FAP in a YTHDF2-dependent manner. Moreover, FTO-mediated metastasis formation depended on the interactions between FAP and integrin family members, which further activated the FAK signaling. Our current findings provided valuable insights into the role of FTO-mediated m^6^A demethylation modification in NSCLC metastasis. FTO was identified as a contributor to NSCLC metastasis through the activation of the FAP/integrin/FAK signaling, which may be a potential therapeutic target for NSCLC.

**Supplementary Information:**

The online version contains supplementary material available at 10.1186/s12964-023-01343-6.

## Introduction

Lung cancer is a malignant tumor, with a high clinical incidence and mortality that rises every year globally [1]. In spite of the advancement of antitumor treatments in non-small cell lung cancer (NSCLC), the prognosis is still unsatisfactory [1]. Tumor metastasis is a significant determinant in the poor prognosis. Most cases (approximately 57%) are in an advanced stage at diagnosis because the early disease is typically asymptomatic [2], and the 5-year relative survival rate of these patients is 6% [3]. The high rate of metastasis and its negative effects on survival highlight a crucial unmet need for a deeper understanding of its pathogenesis and novel treatments.

Cancer cells suffer genetic and epigenetic changes to obtain metastatic competence. N^6^-methyladenosine (m^6^A) modification, or methylation of adenosine at the N^6^ position, is the most universal, abundant, and conserved internal transcript modification, especially in eukaryotic messenger RNA (mRNA) [4, 5]. Methyltransferases, demethylases, and m^6^A binding proteins participate in the process of reversible m^6^A modification of mRNA [4–6]. Dynamic m^6^A modification is vital for many bioprocesses [4, 5], and emerging evidence suggests that m^6^A dysregulation correlates with cancer initiation, progression, metastasis, drug resistance and cancer relapse [5, 7].

Fat mass and obesity-associated protein (FTO) was the first enzyme linked to m^6^A demethylation and was previously considered to be highly associated with fatty acid metabolism [8, 9]. Dysregulation of FTO led to tumorigenesis through an m^6^A-dependent mechanism. FTO is upregulated and exhibits a tumor-promoting role in the majority of cancer types [10], including acute myeloid leukemia (AML) [11], breast cancer [12, 13], liver cancer [14], gastric cancer [15] and colorectal cancer [16]. However, the expression of FTO in NSCLC remains controversial. Shi, Li et al. found that FTO was highly expressed and promoted proliferation by activating KRAS signaling or upregulating the ubiquitin-specific protease 7 (USP7) in an m^6^A-dependent manner in NSCLC [17, 18]. FTO was overexpressed and upregulated myeloid zinc finger protein 1 (MZF1) by decreasing the m^6^A levels, promoting cell proliferation and metastasis in lung squamous cell carcinoma (LUSC) [19]. Highly expressed FTO upregulated cell cycle-related transcription factor-1 (E2F1) by inhibiting the m^6^A modification in NSCLC, hastening the progression of NSCLC [20]. Contrarily, FTO was decreased and negatively associated with poor survival in lung adenocarcinoma (LUAD). The EZH2/β-catenin protein complex, induced by WNT signaling, bound to the LEF/TCF-binding elements at FTO’s promoter region, inhibiting FTO expression level in LUAD [21]. Ning et al. found that lowly expressed FTO in LUAD inhibited human plant homologous finger protein 1 (PHF1) in an m^6^A-YTHDF2-dependent manner, further inhibiting the tumorigenesis of LUAD [22]. This study aimed to investigate FTO expression in NSCLC and its function as an m^6^A eraser in NSCLC metastasis.

Focal adhesion kinase (FAK), a non‐receptor tyrosine kinase, is mainly regulated by integrin signaling. Increased expression levels and/or activation of FAK are found in metastatic human cancers [23]. Zhao et al. have reported that deficiency of the CUL5-SOCS3 complex induced the deposition of integrin β1 and was followed by activation of FAK/SRC signaling, enhancing small cell lung cancer (SCLC) metastasis [24]. Another study revealed that DGKA interacted and activated with the SRC/FAK complex, promoting epithelial-mesenchymal transition (EMT) and angiogenesis, subsequently facilitating NSCLC metastasis [25]. FAK is a key regulator of cancer invasion and metastasis [26]. John C et al. reported that FAK inhibitors could be considered as a potential strategy to combat the acquired resistance of chemotherapy, radiotherapy, targeted therapy, or targeted immune microenvironment therapy [27]. Defactinib (VS6063), a highly effective second-generation FAK inhibitor [28, 29], whose phase II clinical trials in KRAS mutant NSCLC patients have been completed. It was reported that defactinib monotherapy demonstrated modest clinical activity and was generally well tolerated [30].

Here, we reported that upregulation of FTO correlated with metastasis and poor survival in NSCLC. FAK inhibitor defactinib (VS6063) suppressed NSCLC metastasis induced by overexpression of FTO. Mechanistically, FTO facilitated NSCLC metastasis by modifying the m^6^A level of FAP in a YTHDF2-dependent manner. Moreover, FTO-mediated metastasis formation depended on the interactions between FAP and members of the integrin family, which further activated the FAK signaling.

## Methods

### Tissue samples

Forty-four paired NSCLC tissues and para-cancerous tissues were obtained from the First Affiliated Hospital of Soochow University. 24 paired tissues were used for qRT‒PCR and 20 paired tissues were used for western blot analysis. All patients were clinically and pathologically diagnosed with NSCLC according to the Revised International System for Staging Lung Cancer. None of them received either radiotherapy or chemotherapy prior to tissue sampling. Informed consent was obtained from all individual participants and the Ethics Committee of the First Affiliated Hospital of Soochow University approved this study. The corresponding ethical approval code is 2020–375.

### Cell lines and cell culture

NSCLC cell lines (H1299, A549, H1650, H460, HCC827, PC9, SKMES-1) and human immortalized normal epithelial cells (BEAS-2B) were purchased from the Cell Bank of the Chinese Academy of Sciences (Shanghai, China). RPMI 1640 medium or Dulbecco’s modified Eagle’s medium (DMEM) supplemented with 10% fetal bovine serum (Gibco, Carlsbad, CA) was used to cultivate cells, at 37 °C in a 5% CO_2_ atmosphere.

### RNA interference

Small interfering RNAs (siRNAs) specific for the target genes were designed and produced by GenePharma (Shanghai, China). The sequences of the siRNAs could be found in Additional file 1: Table S1. Lipofectamine 2000 (Invitrogen, USA) was used to transiently transfect the indicated cells. Cells were collected after 72 h of transfection.

### Establishment of stable FTO or FAP-overexpressing cell lines

GeneChem Corporation (Shanghai, China) provided overexpression and control lentiviruses. 2 μg/ml puromycin (Sigma‒Aldrich, St. Louis, MO, USA) was used to select cells to establish a stable cell line.

### RNA isolation, cDNA synthesis, and quantitative real‐time PCR (qRT‒PCR) assay

Detailed processes were as we previously described [31]. The primers specific to the target genes are listed in Additional file 1: Table S2.

### Western blot analysis and antibodies

Western blot analysis was conducted according to our previous research [32]. The following antibodies were utilized in this study: anti-FTO (#31687), anti-FAP (#66562), anti-Snail (#3895), anti-Slug (#9585), anti-N-cadherin (#13116), anti-FAK (#13009), anti-p-FAK (Ser397) (#8556), anti-Integrin β1 (#9699) (all from Cell Signaling Technology), anti-Integrin α3 (NBP2-62200) (Novus), anti-YTHDF2 (24744–1-AP, Proteintech). Anti-β-actin (CW0096M), anti-rabbit (CW0103), and anti-mouse (CW0102) secondary antibodies were obtained from Cowin (China).

### Transwell migration and invasion assays

Assays were executed as previously described [32]. Then, the cells were imaged and counted under a microscope (CKX41, Olympus).

### Wound healing assay

Exact steps for the wound healing assay were presented in our earlier study [32]. A microscope (CKX41, Olympus) was used to observe and image cells.

### Human m^6^A epitranscriptomic microarray analysis

Total RNA samples from 6 paired NSCLC tissues and para-cancerous tissues were utilized for the human m^6^A epitranscriptomic microarray analysis. The procedure of m^6^A immunoprecipitation, labeling, hybridization and data analysis were performed as previously described [33, 34].

### Methylated RNA immunoprecipitation (MeRIP) qRT‒PCR

NSCLC cell lines were used to extract total RNA. The Magna MeRIP™ m^6^A kit (A17–10499, Merck Millipore, MA, USA) was used to evaluate m^6^A modification of genes. Enriched m^6^A-modified mRNAs were then detected through qRT‒PCR and the primers were presented in Additional file 1: Table S3.

### RNA-Binding Protein Immunoprecipitation (RIP)

A RIP kit (BersinBio, Guangzhou, China) was used according to the manufacturer’s protocol for RIP analysis. First, NSCLC cells were lysed with RIP lysis buffer. Second, we incubated the lysate products with magnetic beads preconjugated to an anti-IgG, anti-FTO antibody, or anti-YTHDF2 antibody at 4 °C overnight. Then, RNA was extracted and purified by the phenol‒chloroform‒isoamyl alcohol method. Finally, the expression level of FAP was determined by qRT‒PCR.

### Luciferase reporter assays

Assays were executed based on earlier reported [35]. A Dual-Luciferase Reporter Assay Kit (Promega) was used to measure the luciferase activity.

### RNA stability assays

NSCLC cells were treated with actinomycin D (10 mg/ml, Cat#S8964, Selleck, USA) for the indicated times [36]. Total RNA was extracted and processed for qRT‒PCR analysis.

### Co-immunoprecipitation (co-IP) assays

The assays were carried out as previously reported [32]. SDS‒PAGE and western blot analysis were used to separate IgG-bound or FAP-bound proteins.

### In vivo metastasis assays

The Laboratory Animal Center of Soochow University provided female BALB/c nude mice (5 weeks old). Mice were kept under specific pathogen-free conditions. They were injected intravenously (i.v.) with FTO-overexpressing and control A549 cells (1.8 × 10^6^ cells/mouse) to establish the in vivo model of NSCLC metastasis, and were then gavaged with dimethyl sulfoxide (DMSO) (25 mg/kg, daily) or the FAK inhibitor defactinib (VS6063) (Cat#S7654, Selleck, USA) (25 mg/kg, daily) beginning in the fifth week after injection. The mice were euthanized eight weeks after being inoculated, and their lungs were taken out and preserved in Bouin's solution for macroscopic investigation of metastatic nodules. Hematoxylin and eosin (H&E) staining of lung tissues was used to look for micrometastatic foci. The Animal Ethics Committee of Soochow University approved and supervised the animal study.

### Bioinformatics analysis

We conducted differential expression analysis and patient survival analysis with Gene Expression Profiling Interactive Analysis (GEPIA, www.gepia.cancer-pku.cn), the Human Cancer Metastasis Database (HCMDB, http://hcmdb.i-sanger.com/index) or the University of ALabama at Birmingham CANcer data analysis portal (ULCAN, http://ualcan.path.uab.edu). Immunohistochemical staining data were downloaded from the Human Protein Atlas database (https://www.proteinatlas.org). Correlation analysis was performed using the starBase database (https://starbase.sysu.edu.cn). The TCGA database (https://www.cancer.gov) was used to download NSCLC patients’ data. Gene Set Enrichment Analysis (GSEA) 4.1.0 software was used to perform KEGG pathway analysis. We extracted FAP mRNA expression data from the TCGA database to conduct functional analysis using FunRich software (version 3.1.3). We used R software (4.0.3) to analyze data.

### Statistical analysis

The quantitative variables are presented as the mean and SDs. values. Differences between the two groups were assessed using an unpaired Student's t-test. We performed the normality tests before conducting the unpaired Student’s t-test if applicable. GraphPad Prism 8 software (GraphPad, San Diego, CA, USA) was used for statistical analysis. *P* < 0.05 differences were considered significant.

## Results

### High expression of FTO predicts poor prognosis and promotes cell migration and invasion in NSCLC in vitro

**Fig. 1:**
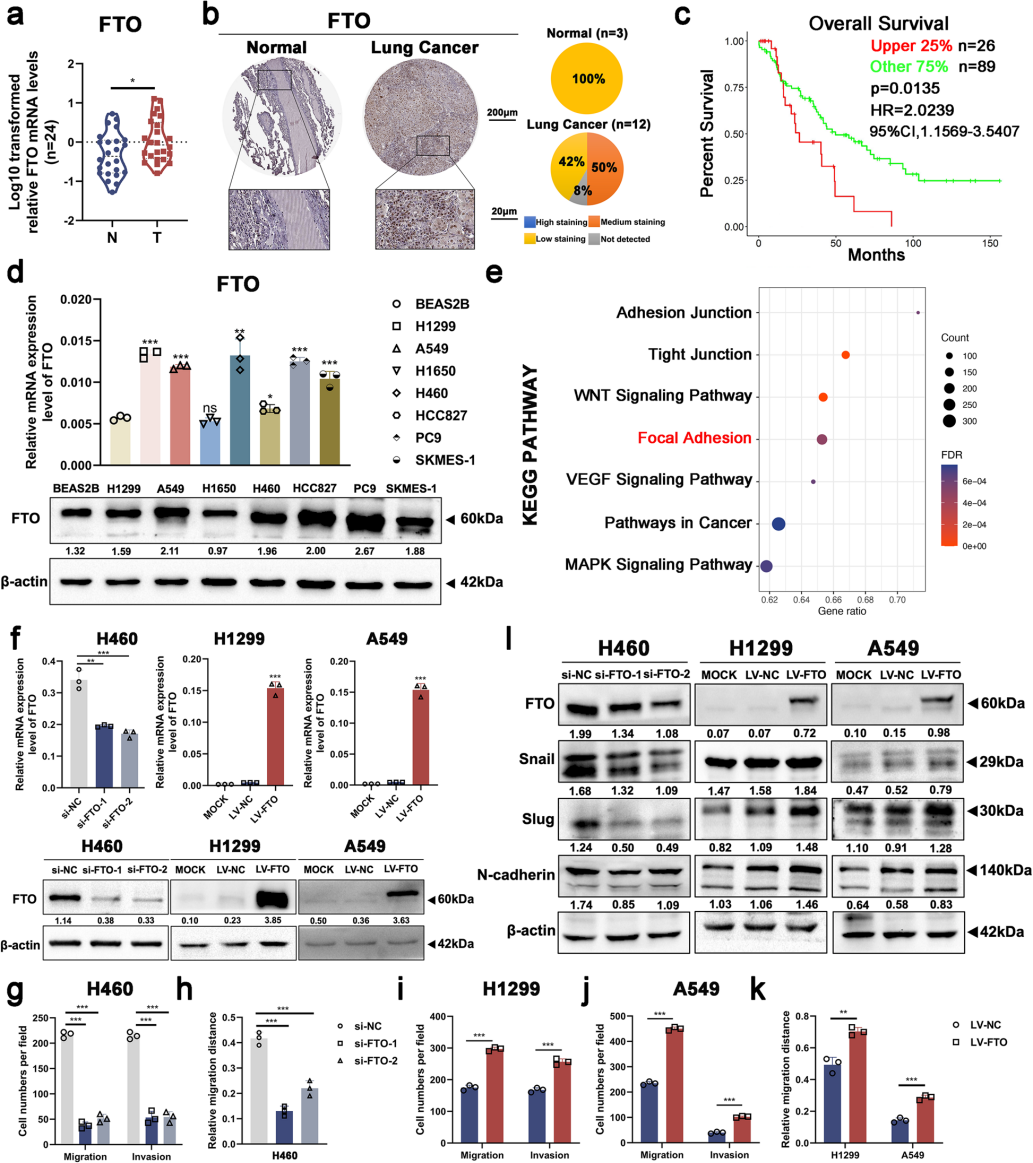
High expression of FTO predicts poor prognosis and promotes cell migration and invasion in NSCLC in vitro. **a** FTO mRNA expression levels were significantly upregulated in 24 NSCLC tissues compared to the paired para-cancerous tissues. **b** Immunohistochemical staining showed cells with moderate FTO staining were observed in NSCLC tissues, while low staining was observed in normal control tissues. Data were collected from the Human Protein Atlas database (https://www.proteinatlas.org). The staining of IHC was clarified as four types (high, medium, low staining or not detected). **c** FTO upregulation was associated with shorter overall survival (OS) times in NSCLC patients (*P *< 0.05). Data were obtained from the GEO database (GSE26939) (https://www.ncbi.nlm.nih.gov/geo/). **d** FTO mRNA and protein expression were significantly elevated in NSCLC cell lines. **e** KEGG analysis reflected that FTO overexpression was related to enrichment of pathways concerning cell metastasis. Data were obtained from the TCGA database. **f** The relative mRNA and protein expression levels of FTO in H460, H1299, and A549 cells. **g-k** Quantitative analysis of Transwell and wound healing assay data in H460, H1299, and A549 cells with FTO knockdown or FTO overexpression. **l** Western blot analysis verified that FTO regulated EMT-related proteins including Snail, Slug, and N-cadherin, in NSCLC. Data information: Data are shown as the mean ± SDs. In all relevant panels, *P < 0.05; **P < 0.01; ***P < 0.001

To examine FTO expression in NSCLC and its potential clinical significance, 44 cases of paired NSCLC tissues and para-cancerous tissues were investigated. qRT‒PCR and western blot results showed a significant increase in FTO mRNA and protein expression levels in NSCLC tissues compared to para-cancerous tissues (Figs. 1a and 3b). Immunohistochemical staining from Human Protein Atlas database (https://www.proteinatlas.org) showed cells with moderate staining of FTO in NSCLC tissues, whereas low staining was observed in normal control tissues (Fig. 1b). In line with the results in NSCLC tissues, FTO mRNA and protein expression levels were significantly elevated in NSCLC cell lines in comparison to BEAS-2B cells (Fig. 1d). Moreover, data from the GEO database (GSE26939) (https://www.ncbi.nlm.nih.gov/geo/) revealed that FTO upregulation was associated with shorter overall survival (OS) times in NSCLC patients (Fig. 1c). KEGG pathway analysis revealed that FTO overexpression was related to enrichment of pathways concerning cell metastasis, including focal adhesion, adhesion junction, and WNT signaling pathway (Fig. 1e). These findings indicated that high expression of FTO predicted poor prognosis and played a pro-metastatic role in NSCLC.

We initially knocked down or stably overexpressed FTO in the H460, H1299, or A549 cell lines to validate its role in cell migration and invasion. These cell lines showed altered FTO mRNA and protein expression levels (Fig. 1f, Figure S1a-c). Transwell and wound healing assays suggested that FTO knockdown inhibited cell migration and invasion (Fig. 1g-h, Figure S1d-i). In contrast, cell migration and invasion were significantly enhanced in FTO-overexpressing cells (Fig. 1i-k, Figure S1j-k). Western blot analysis further verified that FTO could regulate the expression levels of the EMT-related proteins, including Snail, Slug, and N-cadherin in NSCLC (Fig. 1l). Collectively, FTO could promote cell migration and invasion in NSCLC in vitro.

### The FAK inhibitor defactinib (VS6063) can suppress NSCLC metastasis induced by overexpression of FTO in vivo

**Fig. 2:**
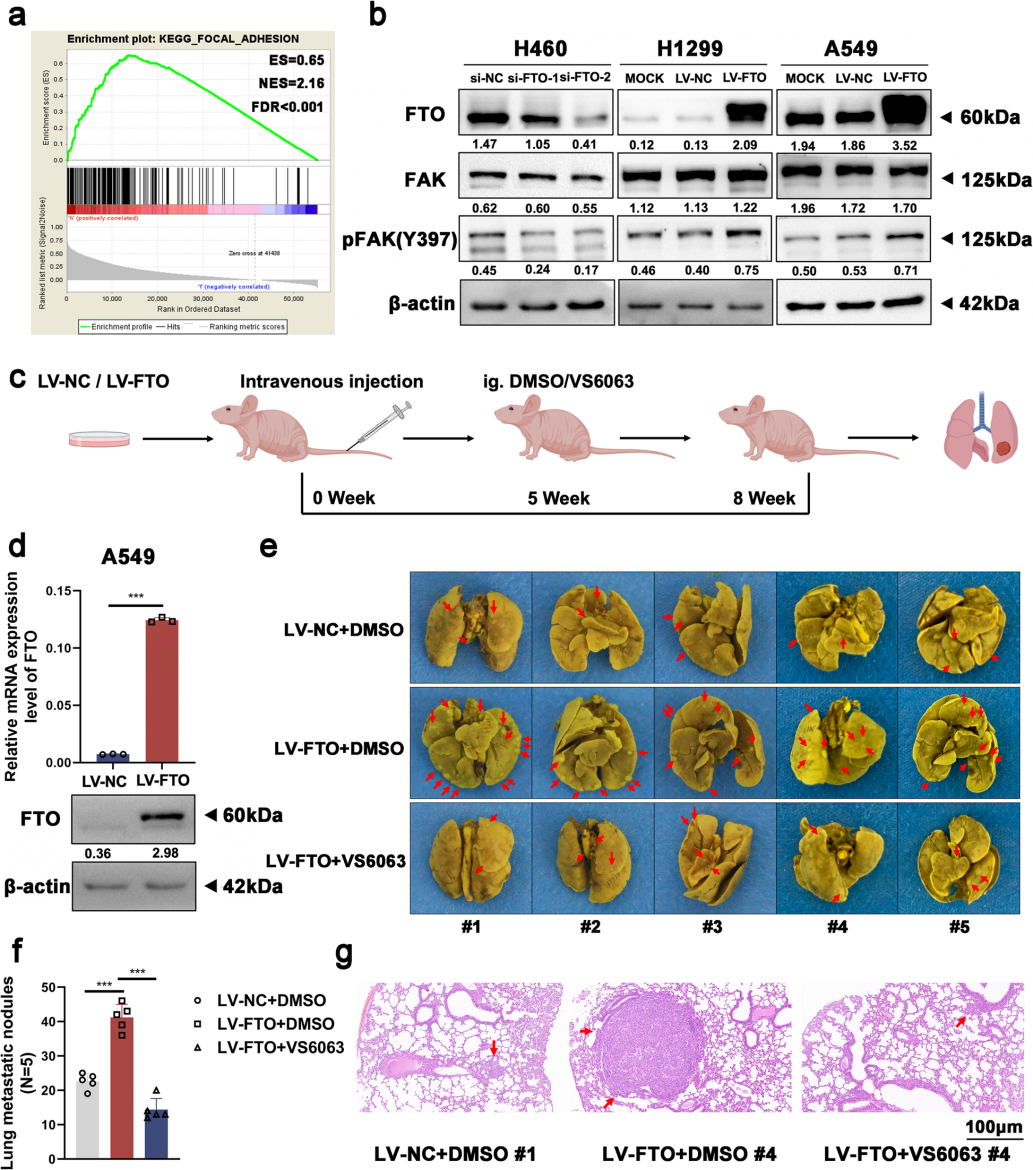
The FAK inhibitor defactinib (VS6063) can suppress NSCLC metastasis induced by overexpression of FTO in vivo. **a** GSEA based on the TCGA dataset showed that high expression of FTO was associated with focal adhesion. ES, enrichment score. NES, normalized enrichment score. FDR, false discovery rate. **b** Western blot analysis showed that FTO can affect FAK signaling. **c** A flowchart of the NSCLC cell in vivo metastasis model. A549 cells overexpressing FTO or empty vector (1.8 × 10^6^ cells/mouse) were injected i.v. into BALB/c nude mice (*n* = 5 mice per group). VS6063 or DMSO was administered i.g. as described in the Methods beginning in week 5. **d** The relative mRNA and protein expression levels of FTO in the A549 cells used to establish the in vivo metastasis model. **e** Photographs of lung metastatic nodules that developed in mice after injection of FTO-overexpressing or control vector A549 cells and administration of DMSO or VS6063. The red arrowheads indicate metastatic nodules that developed in the lungs. **f** Comparison of the number of lung metastatic nodules among the three groups. **g** H&E staining was carried out to evaluate lung micrometastatic foci. Representative histological images of micrometastatic foci in the three groups. The red arrowheads denote micrometastatic foci. Scale bar, 100 μm. Data information: Data are shown as the mean ± SDs. In all relevant panels, *P < 0.05; **P < 0.01; ***P < 0.001

Since it was confirmed that FTO can promote NSCLC cell migration and invasion, the potential mechanism by which FTO facilitates cancer metastasis in NSCLC needed to be further investigated. KEGG pathway analysis revealed an enrichment of the focal adhesion pathway in cells with high expression levels of FTO (Fig. 2a), which suggested that FTO can activate the FAK signaling pathway. Western blot analysis further showed that knockdown of FTO inhibited the expression levels of phosphorylated FAK (p-FAK) and overexpression of FTO significantly increased p-FAK expression levels (Fig. 2b).

Subsequently, we extended our study to the use of the FAK inhibitor and new treatment options for NSCLC metastasis. Defactinib (VS6063) is a selective FAK inhibitor [28]. Both FTO-overexpressing and control cells were treated with DMSO or VS6063 (5 μmol/L) [37] and were used for further analysis. The Transwell and wound healing assays indicated that the FAK inhibitor treatment reversed the excessive cell migration and invasion induced by FTO overexpression (Figure S2a-g).

We then performed in vivo metastasis assays. BALB/c nude mice were injected intravenously (i.v.) with FTO-overexpressing and control A549 cells to establish the in vivo model of NSCLC metastasis and were then given DMSO or the FAK inhibitor defactinib (VS6063) (25 mg/kg, daily), gavage administration, beginning in the fifth week after injection. Eight weeks post inoculation, we euthanized the mice and removed their lungs to assess the metastatic potential (Fig. 2c). The mRNA and protein expression levels of FTO in FTO-overexpressing and control A549 cells were redetermined before inoculation (Fig. 2d). The mice inoculated with FTO-overexpressing A549 cells developed more lung metastatic nodules and micrometastatic foci than those inoculated with control A549 cells (Fig. 2e-g). Moreover, in mice inoculated with FTO-overexpressing A549 cells, fewer lung metastatic nodules and micrometastatic foci were observed in the group treated with VS6063 than in the group treated with DMSO, indicating that the pro-metastatic effect of FTO was blocked by the FAK inhibitor (Fig. 2e-g). Collectively, in vivo experiments showed that the FAK inhibitor defactinib (VS6063) can suppress NSCLC metastasis induced by overexpression of FTO, indicating a novel treatment strategy for NSCLC metastasis.

### FTO modifies the m^6^A level of FAP in an m^6^A-YTHDF2-dependent manner

**Fig. 3:**
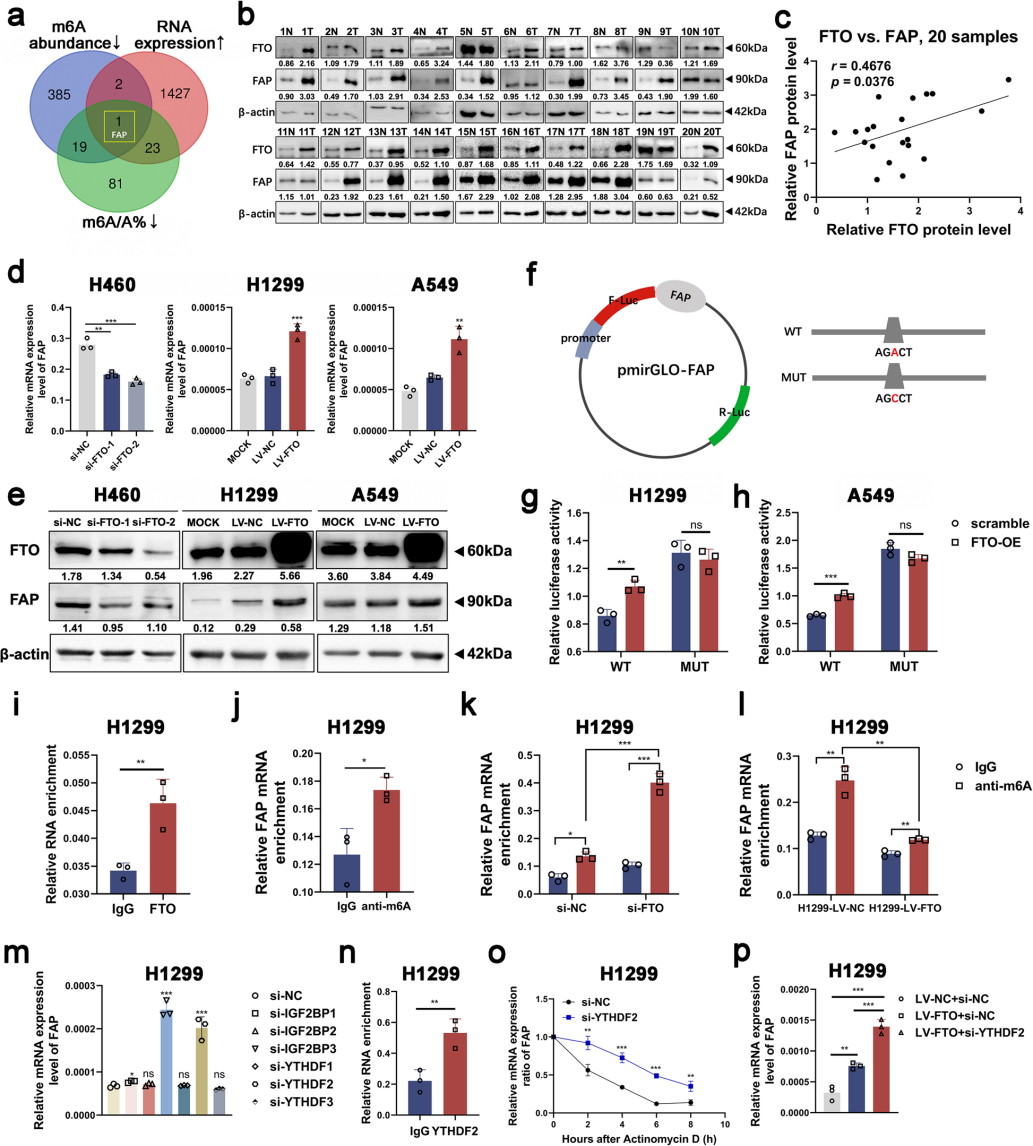
FTO modifies the m^6^A level of FAP in an m^6^A-YTHDF2-dependent manner. **a** FAP was identified as a downstream target of FTO-mediated m^6^A modification by the human m^6^A epitranscriptomic microarray analysis. Venn diagram illustrating the overlap of significantly differentially expressed genes. **b** Western blot assay of FTO and FAP protein expression levels in 20 paired NSCLC tissues and the paired para-cancerous tissues. **c** Correlation analysis of the protein expression levels of FTO and FAP in NSCLC tumor samples. **d** The relative mRNA expression levels of FAP in FTO-knockdown or FTO-overexpressing NSCLC cell lines. **e** The relative protein expression levels of FAP in FTO-knockdown or FTO-overexpressing NSCLC cell lines. **f** Wild-type or m^6^A consensus sequence mutant FAP was fused to a firefly luciferase reporter. Mutation of the m^6^A consensus sequences was generated by replacing adenosine (A) with cytosine (C). **g-h** Relative luciferase activity of the wild-type and mutant FAP reporter vectors in FTO-overexpressing NSCLC cells. **i** RIP assay using an anti-FTO antibody or IgG to detect the binding to FAP in H1299 cells. IgG was used as the negative control. **j-l** MeRIP qRT‒PCR assay, using an anti-m^6^A antibody or IgG to detect the binding to FAP in H1299 cells. IgG was used as the negative control. **m** The mRNA expression levels of FAP were analyzed by qRT‒PCR in H1299 cells transfected with the indicated constructs. **n** RIP assay, using an anti-YTHDF2 antibody or IgG to detect the binding to FAP in H1299 cells. IgG was used as the negative control. **o** The relative mRNA expression ratio of FAP was analyzed by qRT‒PCR in actinomycin D-treated H1299 cells at various time points (2, 4, 6, and 8 h). **p** The relative mRNA expression levels of FAP in H1299 cells transfected with the indicated constructs. Data information: Data are shown as the mean ± SDs. In all relevant panels, *P < 0.05; **P < 0.01; ***P < 0.001

To clarify the specific molecular mechanism of FTO regulating the FAK signaling pathway and identify its downstream targets in NSCLC, a human m^6^A epitranscriptomic microarray was performed to map the m^6^A modifications in NSCLC. The mRNA transcripts with hypomethylated m^6^A peaks are likely potential targets of FTO, since FTO is an m^6^A demethylase. However, there was no significant difference in the m^6^A level of FAK between cancer and para-cancerous tissues. By overlapping the genes with a decreased absolute abundance and percentage of m^6^A modification, as well as the genes with upregulated expression (greater than 1.5-fold change), we identified fibroblast activation protein (FAP) as a potential downstream target of FTO (Fig. 3a). Consistent with the sequencing analysis results, the findings showed a positive correlation between FTO and FAP at both the mRNA and protein expression levels in NSCLC samples, cell lines, and TCGA data (Fig. 3b-e, Figure S3a-c). The RIP assay results indicated that FTO can specifically bind to FAP, which preliminarily verified that FTO served an important role in the m^6^A modification of FAP (Fig. 3i).

To further explore this effect, we performed MeRIP and analyzed the products with qRT‒PCR. We found four presumptive m^6^A modification sites in FAP mRNA using SRAMP [14, 38] (http://www.cuilab.cn/sramp) (Figure S3d) and designed m^6^A specific primers for further MeRIP qRT‒PCR in advance. FAP mRNA was significantly enriched by the anti-m^6^A antibody in H1299 cells in the MeRIP qRT‒PCR assay (Fig. 3j). Furthermore, as expected, the FAP mRNA expression was significantly increased after FTO knockdown, whereas FTO overexpression markedly reduced the FAP mRNA expression (Fig. 3k-l). In addition, MeRIP qRT‒PCR assays showed that site #518 (peak 2) was the most reliably modified region in the FAP transcript (Figure S3e-f). Subsequently, we replaced the N^6^-methylated adenosine (A) with cytosine (C) at site #518 in the m^6^A consensus sequence of FAP mRNA to establish a FAP mutant resistant to m^6^A modification (Fig. 3f). The luciferase reporter assay results showed a significant increase in the luciferase activity of wild-type FAP in FTO-overexpressing cells, while in the mutant cells, the increase was almost completely abolished (Fig. 3g-h), indicating that the modulation of FAP expression was regulated by the FTO-related m^6^A modification at site #518.

Although m^6^A modification can be modulated by “writers” and “erasers”, m^6^A modification operates as a powerful posttranscriptional modulator depending on “readers” in biological processes, who can increase the mRNA translation efficiency or affect mRNA stability after recognizing the m^6^A modification [5, 6]. The expression of FAP rose dramatically as the m^6^A modification levels decreased, indicating that an m^6^A binding protein with a negative effect on methylation is involved in the modification of FAP. Early studies proved that YTHDF2 reduced mRNA stability and promoted targeted mRNA decay [5, 39]. We then knocked down the most common “readers” and tested FAP mRNA expression levels by qRT-PCR. Results showed that knocking down YTHDF2 significantly upregulated FAP mRNA expression in H1299 and A549 cells (Fig. 3m, Figure S4a-c). And in the TCGA database, the expression of FAP was inversely associated with that of YTHDF2 (Figure S4d). IGF2BP3-knockdown cells also showed elevated FAP expression (Fig. 3m, Figure S4a-c), while previous studies have reported that IGF2BP3 was associated with mRNA stabilization and translation [40, 41]. So we hypothesized that YTHDF2, rather than IGF2BP3, participated in the RNA recognition of FAP. Thereafter, we performed RIP assays using antibodies against YTHDF2. The results indicated that YTHDF2 can specifically bind to FAP in both H1299 and A549 cells (Fig. 3n, Figure S4e), which unveiled the crucial role of YTHDF2 in the m^6^A modification of FAP. The stability of FAP mRNA improved after YTHDF2 knockdown in H1299 and A549 cells (Fig. 3o, Figure S4f), revealing that YTHDF2 can facilitate the degradation of FAP mRNA. Moreover, in FTO-overexpressing NSCLC cells, we knocked down YTHDF2, and noticed increased FAP mRNA and protein expression levels in these cells (Fig. 3p, Figure S4g-h). In summary, FTO is thought to increase FAP expression levels by abolishing m^6^A-YTHDF2-dependent mRNA degradation.

### High FAP expression predicts poor prognosis and promotes cell migration and invasion in NSCLC in vitro

**Fig. 4:**
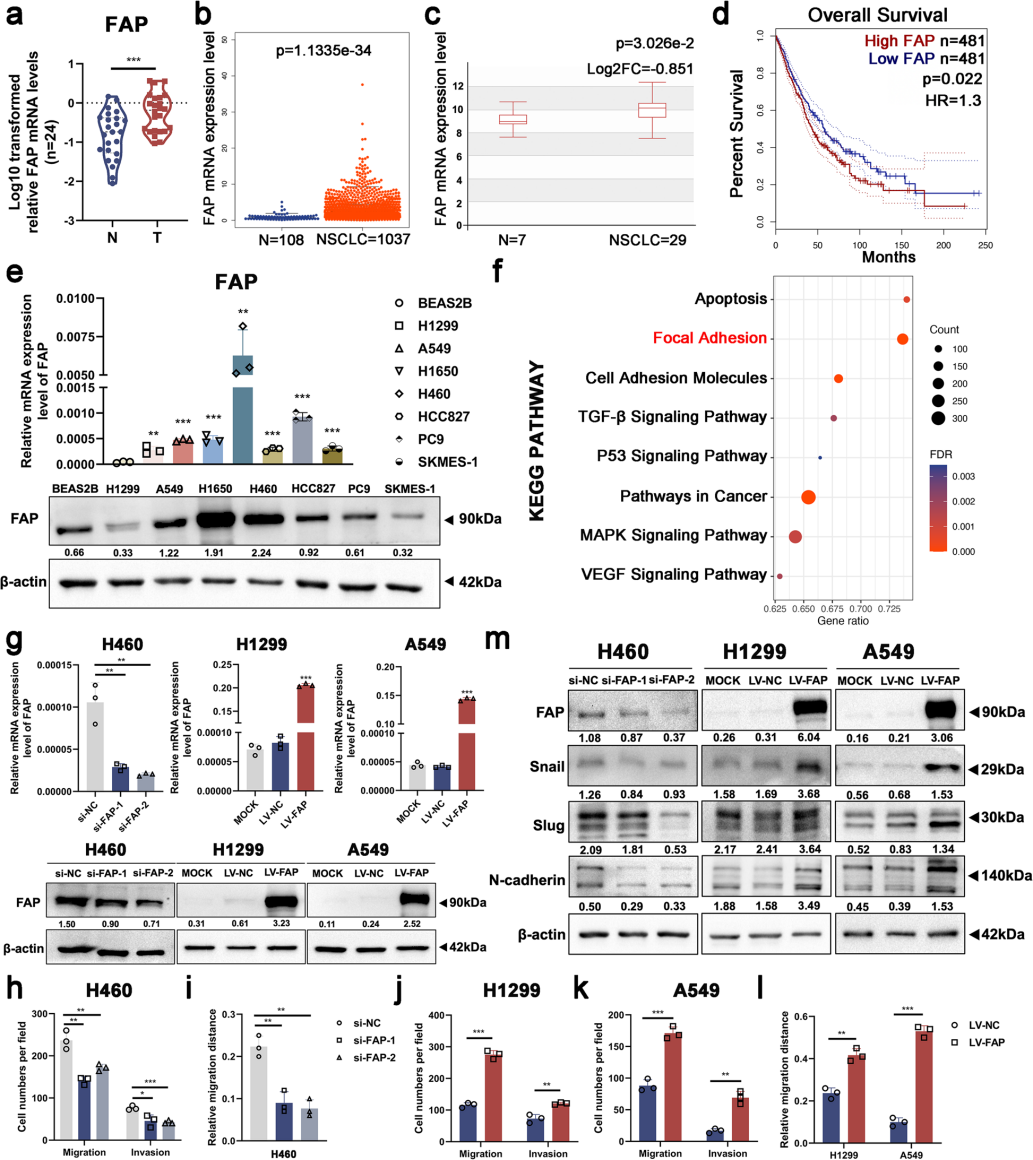
High FAP expression predicts poor prognosis and promotes cell migration and invasion in NSCLC in vitro. **a** The FAP mRNA expression levels were significantly upregulated in 24 NSCLC tissues compared to the paired para-cancerous tissues. **b** FAP mRNA expression levels were significantly upregulated in NSCLC tissues. Data were obtained from the TCGA database. **c** Boxplot of FAP mRNA expression levels in NSCLC and normal tissues. Data were obtained from the HCMDB database (http://hcmdb.i-sanger.com/index). **d** FAP upregulation was associated with shorter overall survival (OS) times in NSCLC patients (*P* < 0.05). Data were obtained from the GEPIA database (http://gepia.cancer-pku.cn). **e** FAP mRNA and protein expression levels were significantly elevated in NSCLC cell lines. **f** KEGG analysis reflected that FAP overexpression is related to enrichment of pathways concerning cell metastasis. Data were obtained from the TCGA database. **g** The relative mRNA and protein expression levels of FTO in H460, H1299, and A549 cells. **h–l** Quantitative analysis of Transwell and wound healing assay data in H460, H1299, and A549 cells with FAP knockdown and FAP overexpression. **m** Western blot analysis verified that FAP regulated EMT-related proteins, including Snail, Slug, and N-cadherin, in NSCLC. Data information: Data are shown as the mean ± SDs. In all relevant panels, *P < 0.05; **P < 0.01; ***P < 0.001

We also detected the expression levels of FAP in the same tissues and cell lines. FAP expression levels were markedly higher in the NSCLC tissues than that in the paired para-cancerous tissues (Figs. 3b and  4a), which was compatible with the results in cell lines (Fig. 4e) and the TCGA and HCMDB database analysis (Fig. 4b-c). Moreover, based on the online UALCAN database, we found that the higher the clinical stage was, the higher the expression levels of FAP (Figure S5a-c). In addition, the Kaplan‒Meier OS curve revealed that upregulation of FAP correlated with poor survival in NSCLC (Fig. 4d). KEGG analysis indicated that FAP overexpression was related to enrichment of pathways concerning cell metastasis, including focal adhesion, cell adhesion molecules, and the TGF-β signaling pathway (Fig. 4f). These findings indicated that high expression of FAP predicts poor prognosis and plays a pro-metastatic role in NSCLC.

To unveil the role of FAP in cell migration and invasion, we successfully constructed FAP-knockdown and FAP-overexpressing H460, H1299, and A549 cell lines, respectively (Fig. 4g). Transwell and wound healing assays were utilized to assess cell migration and invasion abilities. It was demonstrated that cell migration and invasion were suppressed in FAP-knockdown cells (Fig. 4h-i, Figure S5d-e), but enhanced in FAP-overexpressing cells (Fig. 4j-l, Figure S5f-g). The expression levels of EMT-related proteins, including Snail, Slug, and N-cadherin, altered after either knockdown or overexpression of FAP, as shown by western blot analysis (Fig. 4m). In summary, FAP can promote cell migration and invasion in NSCLC in vitro.

### FTO activates the FAK signaling pathway through FAP

**Fig. 5:**
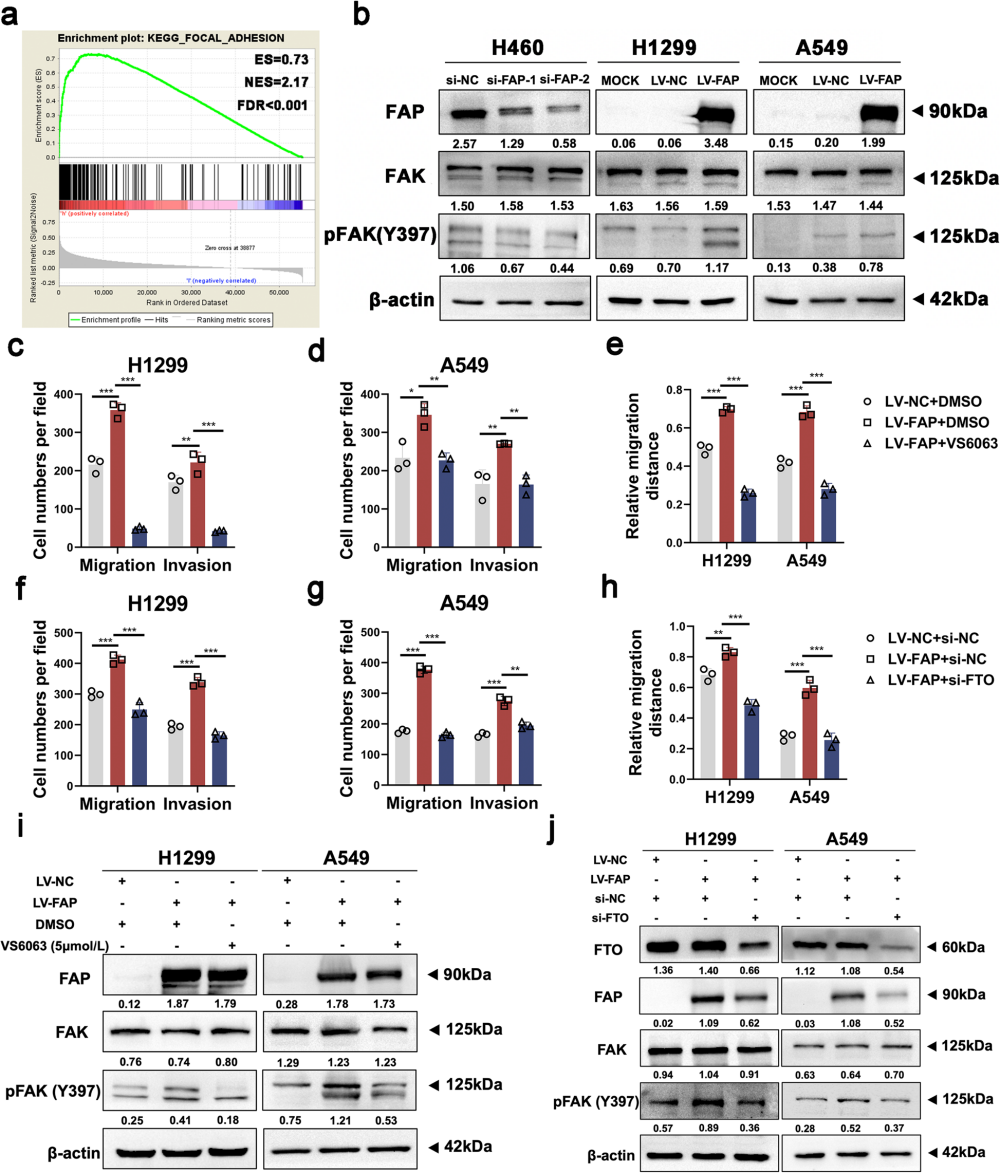
FTO activates the FAK signaling pathway through FAP. **a** GSEA based on the TCGA dataset showed that high expression of FAP was associated with focal adhesion. ES, enrichment score. NES, normalized enrichment score. FDR, false discovery rate. **b** Western blot analysis showed that FAP can affect FAK signaling. **c-e** The FAK-inhibitor defactinib (VS6063) inhibited the FAP-induced cell migration and invasion. Quantitative analysis of Transwell and wound healing assay data in NSCLC cells are shown. **f–h** Knockdown of FTO inhibited the FAP-induced cell migration and invasion. Quantitative analysis of Transwell and wound healing assay data in NSCLC cells are shown. **i** The FAK-inhibitor (VS6063) blocked FAP-induced activation of the FAK signaling pathway in NSCLC cells. **j** Knockdown of FTO rescued FAP-induced activation of the FAK signaling pathway in NSCLC cells. Data information: Data are shown as the mean ± SDs. In all relevant panels, *P < 0.05; **P < 0.01; ***P < 0.001

KEGG pathway analysis also revealed enrichment of the focal adhesion pathway in cells with high FAP expression (Fig. 5a), which suggested that FAP can activate the FAK signaling pathway. The expression levels of p-FAK were significantly changed through FAP knockdown or overexpression (Fig. 5b). We then treated FAP-overexpressing cells with the FAK inhibitor. The expression levels of p-FAK were significantly reduced by VS6063 treatment in the western blot assay (Fig. 5i). Moreover, the Transwell and wound healing assays indicated that the FAK inhibitor reversed the hyperactive cell migration and invasion induced by FAP overexpression (Fig. 5c-e, Figure S6a-d). These findings confirmed that the FAK inhibitor blocked FAP‐induced aberrant activation.

We then performed rescue experiments to establish the existence of the FTO/FAP/FAK regulatory axis in NSCLC. FTO siRNAs were transfected into FAP-overexpressing and control cells, and we identified that knocking down FTO rescued the abnormally altered FAP and p-FAK levels (Fig. 5j). Transwell and wound healing assays also demonstrated that reducing FTO expression reversed the hyperactive cell migration and invasion induced by FAP overexpression (Fig. 5f-h, Figure S6e-h). To sum up, FTO activates the FAK signaling pathway through FAP.

### Integrin signaling is involved in FAP-induced FAK pathway activation

**Fig. 6:**
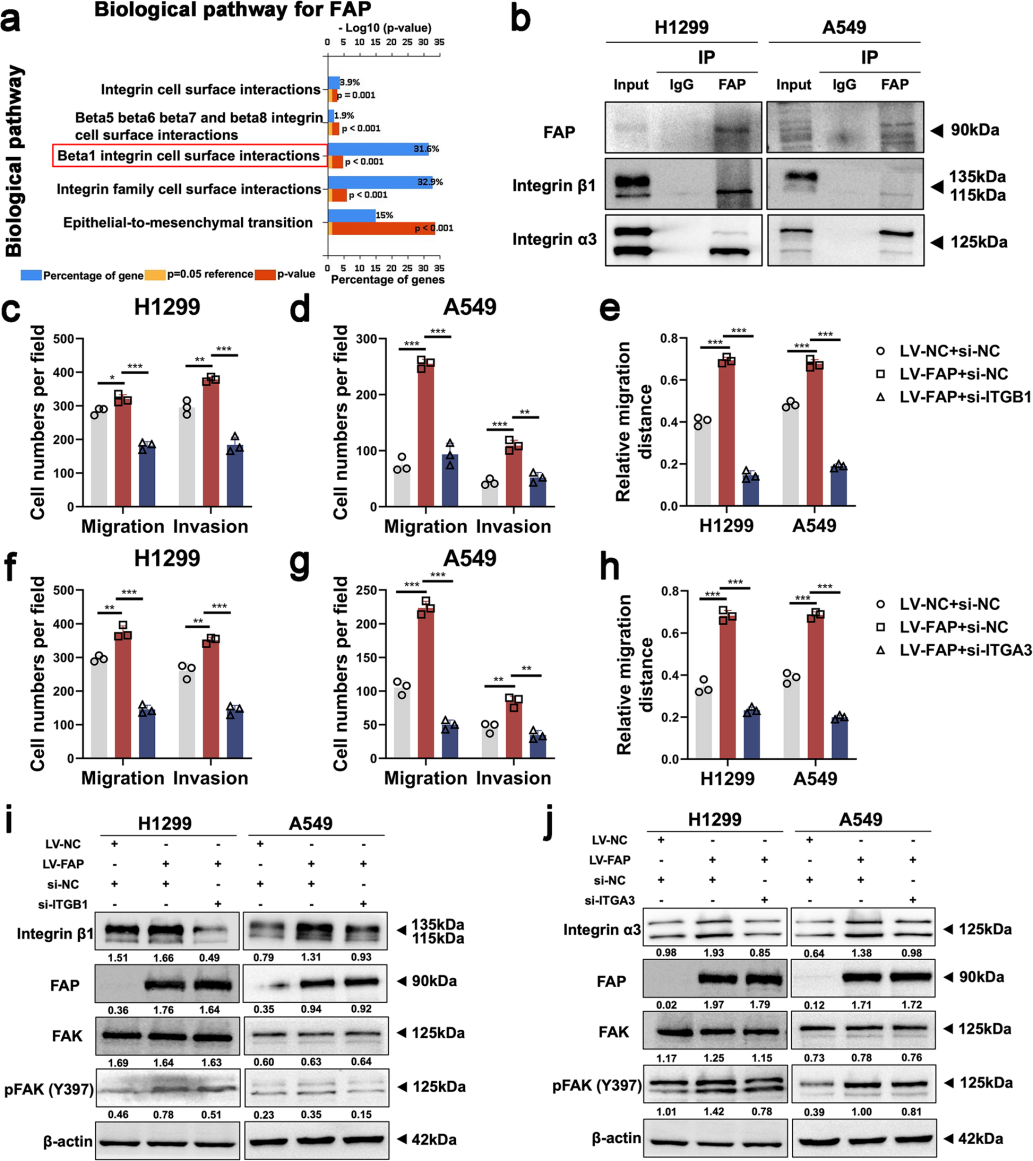
Integrin signaling is involved in FAP-induced FAK pathway activation. **a** Functional analysis of FAP with FunRich software showed that FAP was associated with the integrin family. Data were obtained from the TCGA database. **b** FAP and integrin α3β1 interactions were detected in NSCLC cells by co-IP assays. **c-e** Knockdown of integrin β1 inhibited FAP-induced cell migration and invasion. Quantitative analysis of Transwell and wound healing assay data in NSCLC cells are shown. **f–h** Knockdown of integrin α3 inhibited FAP-induced cell migration and invasion. Quantitative analysis of Transwell and wound healing assay data in NSCLC cells are shown. **i** Knockdown of integrin β1 blocked FAP-induced activation of the FAK signaling pathway in NSCLC cells. **j** Knockdown of integrin α3 blocked FAP-induced activation of the FAK signaling pathway in NSCLC cells. Data information: Data are shown as the mean ± SDs. In all relevant panels, *P < 0.05; **P < 0.01; ***P < 0.001

To further search for the potential molecular mechanism underlying FAP-induced FAK pathway activation, we extracted FAP mRNA expression data from the TCGA database for functional analysis using FunRich software (version 3.1.3). Analysis of the extracted data showed that FAP was closely associated with integrin signaling, especially beta1 integrin cell surface interactions, according to the ordering of the LogFC value from high to low (Fig. 6a). It has been reported that integrin clustering is one of the critical events for the activation of FAK signaling [42]. FAP, as a cell surface serine protease [43], has been reported to interact with integrin α3β1 and the uPAR signaling complex, mediating ovarian cancer cell migration [44]. Therefore, we hypothesized that FAP can interact with integrin α3β1, leading to FAK activation in NSCLC metastasis. We further investigated the direct relationships between FAP and integrins using coimmunoprecipitation (co-IP), and the results showed that FAP specifically interacted with integrin α3β1 in NSCLC cells (Fig. 6b). Then, we found by Transwell and wound healing assays that knockdown of integrin β1 and integrin α3 inhibited the excessive cell migration and invasion induced by FAP overexpression (Fig. 6c-h, Figure S7a-h). In addition, knockdown of integrin β1 and integrin α3 reduced the abnormal increase in the expression levels of p-FAK induced by FAP, as determined by western blot analysis (Fig. 6i-j). These results revealed that FAP activates the FAK signaling pathway by interacting with integrin α3β1.

## Discussion

**Fig. 7:**
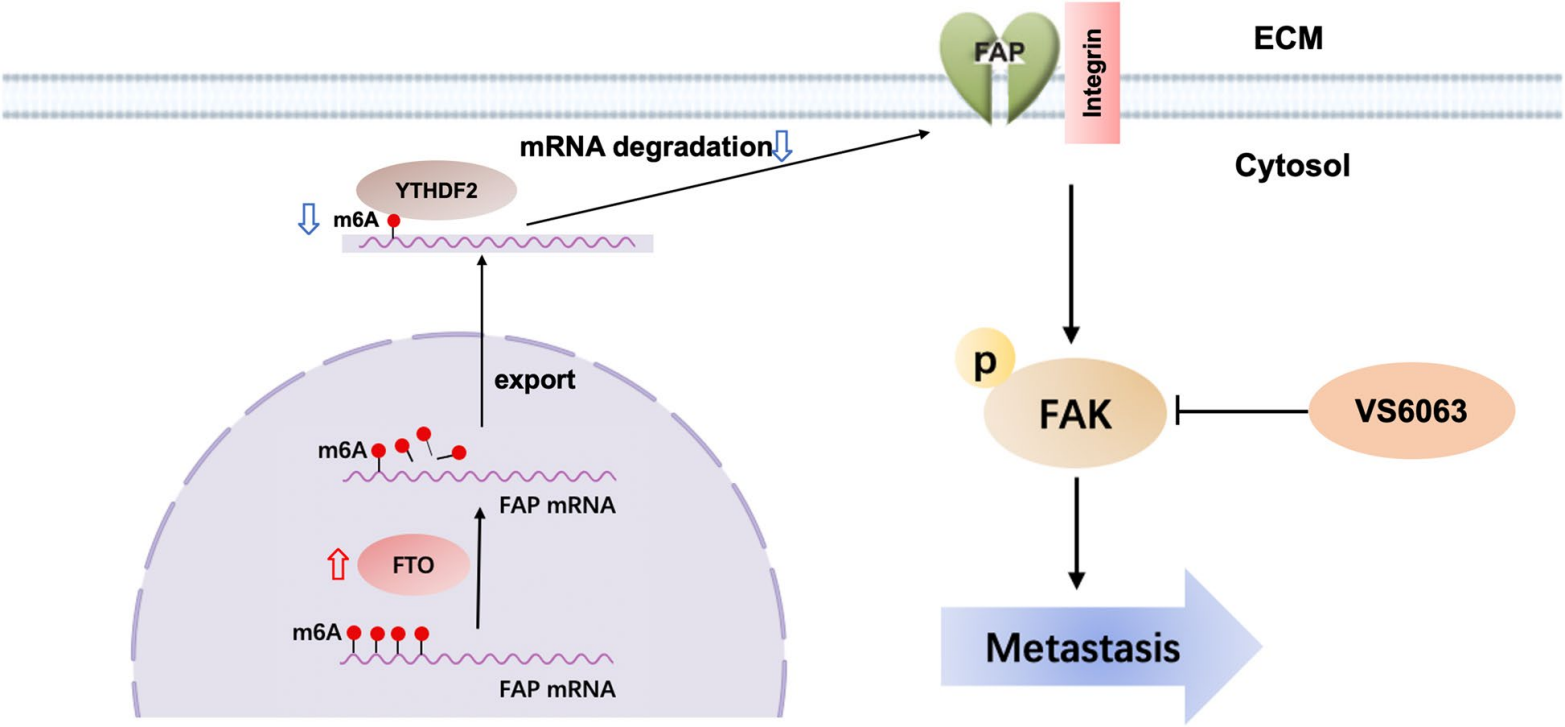
Working model. FTO was upregulated and predicted poor prognosis in patients with NSCLC. FTO promoted cell migration and invasion in NSCLC, and the FAK inhibitor defactinib (VS6063) suppressed NSCLC metastasis induced by overexpression of FTO. Mechanistically, FTO facilitated NSCLC metastasis by modifying the m^6^A level of FAP in an m^6^A-YTHDF2-dependent manner. Moreover, FTO-mediated metastasis formation depended on the interactions between FAP and integrin family members, which further activated the FAK signaling

Tumor metastasis is a major factor contributing to poor prognosis and an ongoing challenge in NSCLC therapy. Accumulating evidence indicates that epigenetic modifications, such as m^6^A, participate in some biological processes in malignant tumor development [5, 39, 45]. As a main demethylase of m^6^A, FTO reduces the levels of m^6^A modification and has been reported to promote cell proliferation in NSCLC [17–19]. However, the function of FTO as an m^6^A eraser in NSCLC metastasis remains unknown. Here, we demonstrated that FTO was upregulated and predicted poor prognosis in patients with NSCLC. FTO promoted cell migration and invasion in NSCLC, and the FAK inhibitor defactinib (VS6063) suppressed NSCLC metastasis induced by overexpression of FTO. Mechanistically, FTO facilitated NSCLC metastasis by modifying the m^6^A level of FAP in a YTHDF2-dependent manner. Moreover, FTO-mediated metastasis formation depended on the interactions between FAP and integrin family members, which further activated FAK signaling (Fig. 7).

We first detected the expression levels of FTO in NSCLC and found that FTO was upregulated in NSCLC. This finding was consistent with previous studies [17–19]. We found that FTO enhanced the ability of cell migration and invasion and regulated EMT-related proteins in NSCLC. EMT has been linked to carcinogenesis and gives cancer cells metastatic capabilities by promoting invasion and resistance to apoptotic stimuli [46]. Hence FTO exerted oncogenic effects on NSCLC metastasis. Moreover, FTO activated the FAK signaling pathway, and the pro-metastatic effects of FTO were suppressed by defactinib (VS6063), a selective FAK inhibitor [28], indicating a novel treatment strategy for NSCLC metastasis.

The role of FTO in NSCLC is conflicting, as different studies have reported opposite effects [17–22]. It is not unusual that an m^6^A regulator has dual roles even in the same cancer. In glioblastoma, the METTL3-METTL14 complex was discovered to be both tumor-promoting and tumor-suppressive [47]. Cui et al. showed that METTL3 and METTL14 acted as tumor suppressors. Key oncogenic transcripts like a disintegrin and metalloproteinase domain­containing protein 19 (ADAM19) were less stable due to m^6^A methylation [48]. Conversely, METTL3 facilitated cell survival by stabilizing the mRNA of SRY­box 2 (SOX2) in glioblastoma [49]. Another publication confirmed the METTL3-METTL14 complex’s tumorigenic function through the stabilization of mRNAs encoding splicing factors in glioblastoma [50]. Similarly, despite the fact that most studies have demonstrated that ALKBH5 is an oncogene in NSCLC [51–53], another work reported its tumor-suppressive role in NSCLC [54]. ALKBH5 restrained tumor growth and metastasis by downregulating YTHDFs-mediated YAP expression and suppressing miR-107/LATS2–mediated YAP activity in NSCLC [54, 55]. Taken together, it's probable that the disparate conclusions indicate the heterogeneity of human malignancies. The distinct functions of FTO observed in NSCLC may be due to the different subtypes utilized and the genetic heterogeneity in the specific NSCLC tissues [47]. Moreover, given the fact that FTO posttranscriptionally modulates a large variety of target genes, either in a positive or negative way, participating in the cellular processes and determining the fate of tumor cells. Therefore, FTO’s impact on cancer is primarily determined by its functional targets in a specific cancer type or cellular context [55]. Certainly, more research is needed to fully comprehend the function of FTO in NSCLC.

We then sought to elucidate the specific molecular mechanism by which FTO regulates the FAK signaling pathway. Since FTO is the major m^6^A demethylase, the upregulation of FTO implies aberrant m^6^A modifications. Analysis of the human m^6^A epitranscriptomic microarray identified FAP as a downstream target of FTO-mediated m^6^A modification, which was further confirmed by RIP, MeRIP qRT‒PCR, and luciferase reporter assays. An m^6^A reader is required to decide the fate of methylated RNA for its further functions [5]. YTHDF2 was proven through RIP and qRT‒PCR assays to be the reader participating in the FTO-mediated demethylation of FAP. It has been reported that YTHDF2 selectively binds to m^6^A-containing mRNA and localizes the YTHDF2-mRNA complex to sites of cellular RNA decay to promote mRNA degradation [39, 56]. Consistent with this finding, the RNA stability assays in our study showed that the stability of FAP mRNA was significantly improved after YTHDF2 knockdown. We also observed higher expression levels of FAP in FTO-overexpressing NSCLC cells after YTHDF2 knockdown. To sum up, FTO regulates the demethylation of FAP in an m^6^A-YTHDF2-dependent manner.

FAP is a cell surface serine protease [43] and is crucial in extracellular matrix (ECM) remodeling, intracellular signaling regulation, angiogenesis, EMT and immunosuppression [57]. FAP is highly expressed in a wide range of malignancies and is associated with worse clinical outcomes [43, 44, 57]. In the SK-MES-1 cell line, FAP overexpression can enhance cell proliferation, motility, and invasion while also activating PI3K/Akt and SHH/Gli1 signaling [58]. In our study, FAP was highly associated with NSCLC metastasis, as indicated by its higher expression levels in samples with metastasis. Its pro-metastatic role in NSCLC cells was further confirmed by Transwell and wound healing assays. Our KEGG pathway analysis and western blot analysis also revealed that FAP can activate the FAK signaling pathway, and that inhibition of FAK signaling blocks FAP‐induced aberrant activation.

However, how FAP induces FAK signaling in NSCLC was still unknown. We thus conducted functional analysis and found that FAP was closely associated with beta1 integrin cell surface interactions. The proper localization of FAP to invadopodia requires integrin α3β1 [59]. Moreover, Yang et al. further demonstrated that FAP-integrin dimer formation promoted proliferation and migration, while integrin inhibition reversed this phenotype in ovarian cancer [44]. In our study, co-IP assays illustrated the interaction between FAP and integrin α3β1, and inhibition of integrin reversed the phenotype in NSCLC cell lines. These results revealed that FAP can induce cell migration and invasion through interaction with integrin α3β1 in NSCLC. A large body of literature has characterized integrin functions in tumor metastasis [60]. Integrin clustering is one of the critical events for the activation of FAK signaling [42, 60]. Western blot analysis further showed that the knockdown of integrin β1 and integrin α3 reduced the abnormal increase in the expression levels of p-FAK induced by FAP. Based on this evidence, we concluded that FAK signaling was the downstream signaling induced by the FAP-integrin complex in NSCLC metastasis. Furthermore, we performed rescue experiments, which strongly confirmed that FTO plays a pro-metastatic role by upregulating FAP to activate the FAK signaling pathway in NSCLC.

While it is true that several studies have reported that FTO could regulate other genes in an m^6^A-dependent manner [12, 13, 38, 61–65]. There are several important distinctions that set our study apart and contribute to its novelty. First, we believe the role of FTO as an m^6^A eraser in NSCLC metastasis still needs to be verified due to the heterogeneity of different types of cancers. Our research is the first to investigate the regulatory relationship between FTO and FAP. And we found that FTO/m^6^A/FAP axis activated the FAK signaling, depending on the interactions between FAP and integrin family members, which further promoted NSCLC metastasis. Moreover, our research reported that FAK inhibitor defactinib (VS6063) suppressed NSCLC metastasis induced by overexpression of FTO. Although there exist FTO inhibitors used in cancer research, such as R-2-hydroxyglutarate (R-2HG) [66, 67] and FB23-2 [68, 69], it is still in the preclinical research stage. The FAK inhibitor, VS6063, has completed the phase II clinical trials and showed monotherapy demonstrated modest clinical activity and was generally well-tolerated [30], providing a more feasible treatment for NSCLC metastasis. Finally, our study incorporates methodological refinements and improvements. MeRIP, RIP and luciferase reporter assays were used to explore the mechanism of FTO action. RIP assays and RNA stability assays were used to recognize the m^6^A binding proteins involved in FAP demethylation modification. Bioinformatics analyses and co-IP assays were used to determine the specific mechanism of FTO/FAP axis in NSCLC metastasis. These methodological advancements contribute to the reliability and robustness of our findings.

There were still limitations of this study. Since our study demonstrated the important role of the FTO/FAP/FAK axis in NSCLC metastasis, targeting the FTO/FAP/FAK axis with selective inhibitors may be an appealing therapeutic focus for NSCLC patients. R-2-hydroxyglutarate (R-2HG) and FB23-2, inhibitors of FTO, have exhibited intrinsic antitumor activity in AML [67, 69, 70]. Moreover, using boronic acid-based compounds, Poplawski et al. discovered potent FAP-selective inhibitors in recent times [71]. However, the role of FTO or FAP inhibitors in NSCLC remains unknown. We shall find solutions to these issues in further investigations.

## Conclusion

Our current findings provided valuable insights into the role of FTO-mediated m^6^A demethylation in NSCLC metastasis. FTO was identified as a contributor to NSCLC metastasis through the activation of FAP/integrin/FAK signaling, which could be a promising target for NSCLC treatment.

### Supplementary Information

**Additional file 1: Table S1.** Sequences of siRNAs. **Table S2.** Sequences of Primers for Real-time Polymerase Chain Reaction. **Table S3.** Sequences of Primers for MeRIP. 

**Additional file 2:** **Figure S1.** FTO promotes cell migration and invasion in NSCLC *in vitro.* a-b The relative mRNA expression levels of FTO in H1299 and A549 cells after transfection with siRNA. c The relative protein expression levels of FTO in H1299 and A549 cells after transfection with siRNA. d-e Representative images of the Transwell cell migration and invasion assays in H460 and A549 cells (si-FTO compared with si-NC). f-g Wound healing assays were performed to evaluate the role of FTO in H460 and A549 cells (si-FTO compared with si-NC). h-i Quantitative analysis of Transwell and wound healing assay data in FTO-knockdown A549 cells. j Representative images of the Transwell cell migration and invasion assays in H1299 and A549 cells (LV-FTO compared with LV-NC). k Wound healing assays were performed to evaluate the role of FTO in H1299 and A549 cells (LV-FTO compared with LV-NC). Data information: Data are shown as the mean ± SDs. In all relevant panels, *P < 0.05; **P < 0.01; ***P< 0.001.

**Additional file 3:** **Figure S2.** The FAK inhibitor defactinib (VS6063) inhibits the FTO-induced cell migration and invasion. a-b Representative images of the Transwell cell migration and invasion assays in H1299 and A549 cells. c-d Wound healing assays were performed to evaluate the role of VS6063 in H1299 and A549 cells. e-g Quantitative analysis of Transwell and wound healing assay data in H1299 and A549 cells. Data information: Data are shown as the mean ± SDs. In all relevant panels, *P < 0.05;**P < 0.01; ***P < 0.001.

**Additional file 4:** **Figure S3.** FTO modifies the m^6^A level of FAP. a Correlation analysis of the mRNA expression levels of FTO and FAP in NSCLC. Data were obtained from starBase database (https://starbase.sysu.edu.cn). b Relative quantification of FTO and FAP protein expression levels in 20 paired NSCLC tissues and adjacent tissues. The Y-axis shows the log10-transformed fold change in the T/N expression ratio. The X-axis shows the sample number. c Correlation analysis of the mRNA expression levels of FTO and FAP in NSCLC tumor samples based on qRT‒PCR. d M^6^A modification site prediction. Four m^6^A sites in FAP were predicted with SRAMP (http://www.cuilab.cn/sramp). e MeRIP qRT‒PCR assay, using an anti-m^6^A antibody or IgG to detect the binding to FAP in H1299 cells by 4 primers. IgG was used as the negative control. f MeRIP qRT‒PCR assay, using an anti-m^6^A antibody or IgG to detect the binding to FAP in A549 cells by 4 primers. IgG was used as the negative control. Data information: Data are shown as the mean ± SDs. In all relevant panels, *P < 0.05; **P < 0.01; ***P< 0.001.

**Additional file 5:** **Figure S4. **YTHDF2 is involved in FTO-mediated m^6^A demethylation modification in NSCLC. a-b The relative mRNA expression levels of the indicated genes in H1299 and A549 cells after transfection with the indicated constructs. c The mRNA expression levels of FAP were analyzed by qPCR in A549 cells transfected with the indicated constructs. d Correlation analysis of the mRNA expression levels of YTHDF2 and FAP in NSCLC. Data were obtained from the starBase database (https://starbase.sysu.edu.cn). e RIP assays, using an anti-YTHDF2 antibody or IgG to detect the binding to FAP in A549 cells. IgG was used as the negative control. f The relative mRNA expression ratio of FAP was analyzed by qPCR in actinomycin D-treated A549 cells at various time points (2, 4, 6, and 8 hours). g The relative mRNA expression levels of FAP in A549 cells transfected with the indicated constructs. h Western blot analysis verified the increased protein expression levels of FAP in FTO-overexpressing NSCLC cells after YTHDF2 knockdown. Data information: Data are shown as the mean ± SDs. In all relevant panels, *P< 0.05; **P < 0.01; ***P < 0.001.

**Additional file 6:** **Figure S5.** FAP promotes cell migration and invasion in NSCLC *in vitro.* a-b FAP mRNA expression levels were significantly upregulated in LUAD and LUSC, and the higher the clinical stage was, the higher the expression levels of FAP. Data were obtained from the online UALCAN database (http://ualcan.path.uab.edu). c The FAP protein expression levels were significantly upregulated in LUAD, the higher the clinical stage was, the higher the expression levels of FAP. Data were obtained from the online UALCAN database (http://ualcan.path.uab.edu). d Representative images of the Transwell cell migration and invasion assays in H460 cells (si-FAP compared with si-NC). e Wound healing assays were performed to evaluate the role of FAP in H460 cells (si-FAP compared with si-NC). f Representative images of the Transwell cell migration and invasion assays in H1299 and A549 cells (LV-FAP compared with LV-NC). g Wound healing assays were performed to evaluate the role of FAP in H1299 and A549 cells (LV-FAP compared with LV-NC). h Long-exposure images of FAP protein bands in Fig. 4. 

**Additional file 7:** **Figure S6.** The FAK inhibitor defactinib (VS6063) inhibits the FAP-induced cell migration and invasion and rescue experiments. a-b Representative images of the Transwell cell migration and invasion assays in H1299 and A549 cells. c-d Wound healing assays were performed to evaluate the role of VS6063 in H1299 and A549 cells. e-f Representative images of the Transwell cell migration and invasion assays in H1299 and A549 cells. g-h Wound healing assays were performed in H1299 and A549 cells.

**Additional file 8:** **Figure S7.** Integrin signaling is involved in FAP-induced FAK pathways activation. a-b, e-f Representative images of the Transwell cell migration and invasion assays in H1299 and A549 cells. c-d, g-h Wound healing assays were performed in H1299 and A549 cells.

## Data Availability

All data generated or analyzed during this study are included in this published article and its supplementary information files.

## Abbreviations

AML: Acute myeloid leukemia
ADAM19: A disintegrin and metalloproteinase domain­containing protein 19
co-IP: Co-immunoprecipitation
DMEM: Dulbecco’s modified Eagle’s medium
DMSO: Dimethyl sulfoxide
ECM: Extracellular matrix
EMT: Epithelial-mesenchymal transition
E2F1: Cell cycle-related transcription factor-1
FAK: Focal adhesion kinase
FAP: Fibroblast activation protein
FTO: Fat mass and obesity-associated protein
GEPIA: Gene Expression Profiling Interactive Analysis
GSEA: Gene Set Enrichment Analysis
HCMDB: Human cancer metastasis database
LUAD: Lung adenocarcinoma
LUSC: Lung squamous cell carcinoma
m^6^A: N^6^-methyladenosine
MeRIP: M^6^A-RNA immunoprecipitation
METTL3: Methyltransferase-like 3
METTL14: Methyltransferase-like 14
mRNA: Messenger RNA
MZF1: Myeloid zinc finger protein 1
NSCLC: Non‐small cell lung cancer
OS: Overall survival
p-FAK: phosphorylated FAK
PHF1: Plant homologous finger protein 1
qRT‒PCR: Quantitative real-time PCR
RIP: RNA immunoprecipitation
R-2HG: R-2-Hydroxyglutarate
SCLC: Small cell lung cancer
siRNA: Small interfering RNA
TCGA: The Cancer Genome Atlas Program
ULCAN: The University of ALabama at Birmingham CANcer data analysis portal
USP7: Ubiquitin-specific protease 7
