# Supplementary material for: FTO facilitates cancer metastasis by modifying the m6A level of FAP to induce integrin/FAK signaling in non-small cell lung cancer
Source: Cell Commun Signal. 2023 Nov 2;21:311. doi: 10.1186/s12964-023-01343-6 (PMC10623768; doi:10.1186/s12964-023-01343-6)
Supplement: Supplementary file 2 — Additional file 1: Table S1. Sequences of siRNAs. Table S2. Sequences of Primers for Real-time Polymerase Chain Reaction. Table S3. Sequences of Primers for MeRIP. [file 12964_2023_1343_MOESM1_ESM.docx]

Additional file 1

Table S1. Sequences of siRNAs.

| siRNA | Sense | Anti-sense |
| --- | --- | --- |
| si-NC | 5′-UUCUCCGAACGUGUCACGUTT-3′ | 5′-ACGUGACACGUUCGGAGAATT-3′ |
| si-FTO-1 | 5′-AGCUAAAUAUCCUAAACUTT-3′ | 5′-AGUUUAGGAUAUUUCAGCUGC-3′ |
| si-FTO-2 | 5′-GUGGCAGUGUACAGUUAUATT-3′ | 5′-UAUAACUGUACACUGCCACTT-3′ |
| si-FAP-1 | 5′-GAUGAUUCUUCCUCCUCAATT-3′ | 5′-UUGAGGAGGAAGAAUCAUCTT-3′ |
| si-FAP-2 | 5′-CGCCCUUCAAGAGUUCAUATT-3′ | 5′-UAUGAACUCUUGAAGGGCGTT-3′ |
| si-YTHDF1-1 | 5′-GGACAGUCAAAUCAGAGUA-3′ | 5′-UACUCUGAUUUGACUGUCC-3′ |
| si-YTHDF2-1 | 5′-GCUCUGGAUAUAGUAGCAATT-3′ | 5′-UUGCUACUAUAUCCAGAGCTT-3′ |
| si-YTHDF3-1 | 5′-AGUCUGUUGUGAACUAUAATT-3′ | 5′-UUAUAGUCCACAACAACUUC-3′ |
| si-IGF2BP1-1 | 5′-GGAAAUAAUGAAGAAAGUUCGTT-3′ | 5′-CGAACUUUCUUCAUUAUUUCCTT-3′ |
| si-IGF2BP2-1 | 5′-GAGAUAGAGAUUAUGAAGATT-3′ | 5′-UCUUCAUAAUCUCUAUCUCTT-3′ |
| si-IGF2BP3-1 | 5′-GGUGAAUGAACUUCAGAAUTT-3′ | 5′-AUUCUGAAGUUCAUUCACCTT-3′ |
| si-ITGB1-1 | 5′-CAGCCCAUUUAGCUAAAAT-3′ |  |
| si-ITGA3-1 | 5′-UUACAGAGACUUUGACCGATT-3′ |  |

Table S2. Sequences of Primers for Real-time Polymerase Chain Reaction.

| Gene | Forward | Reverse |
| --- | --- | --- |
| FTO | 5′-ACTTGGCTCCCTTATCTGACC-3′ | 5′-TGTGCAGTGTGAGAAAGGCTT-3′ |
| FAP | 5′- ATCTATGACCTTAGCAATGGAGAATTTGT -3′ | 5′- GTTTTGATAGACATATGCTAATTTACTCCC -3′ |
| YTHDF1 | 5′-ACCTGTCCAGCTATTACCCG-3′ | 5′-TGGTGAGGTATGGAATCGGAG-3′ |
| YTHDF2 | 5′-AGCCCCACTTCCTACCAGATG-3′ | 5′-TGAGAACTGTTATTTCCCCATGC-3′ |
| YTHDF3 | 5′-GCTCCACCAACCCAACCAGTTC-3′ | 5′-CTGAGGTCCTTGTTGCTGCTGTG-3′ |
| IGF2BP1 | 5′-GATGAAGGCCATCGAAACTTTC-3′ | 5′-GGGGTGGAATATTTCGGATTTG-3′ |
| IGF2BP2 | 5′-ATCGGGAGCAAACCAAAGACCATC-3′ | 5′-CTGGCAAACCTGGCTGACCTTC-3′ |
| IFG2BP3 | 5′-GAGGCGCTTTCAGGTAAAATAG-3′ | 5′-AATGAGGCGGGATATTTCGTAT-3′ |
| β-actin | 5′-CCTGGCACCCAGCACAAT-3′ | 5′-GGGCCGGACTCGTCATAC-3′ |

Table S3. Sequences of Primers for MeRIP.

|  | Forward | Reverse |
| --- | --- | --- |
| FAP m^6^A peak1 (#2003) | 5′-ATGTAGACTATCTTCTCATCCACGG-3′ | 5′-CTGCTTTAGGAAGTGGGTCATG-3′ |
| FAP m^6^A peak2 (#518) | 5′-GTATTTATGCTGGTCGCCTGTTG-3′ | 5′-ATTTCCTCTTCATAAACCCAGTCTG-3′ |
| FAP m^6^A peak3 (#955) | 5′-GATAGCCTCAAGTGATTATTATTTCAGTTGGC-3′ | 5′- CAGCCCATCCAGTTCTGCTTTCTTC-3′ |
| FAP m^6^A peak4 (#1837) | 5′- ATCTATGACCTTAGCAATGGAGAATTTGT -3′ | 5′-GTTTTGATAGACATATGCTAATTTACTCCC -3′ |
