# Supplementary figures and images for: FTO facilitates cancer metastasis by modifying the m6A level of FAP to induce integrin/FAK signaling in non-small cell lung cancer
Source: Cell Commun Signal. 2023 Nov 2;21:311. doi: 10.1186/s12964-023-01343-6 (PMC10623768; doi:10.1186/s12964-023-01343-6)

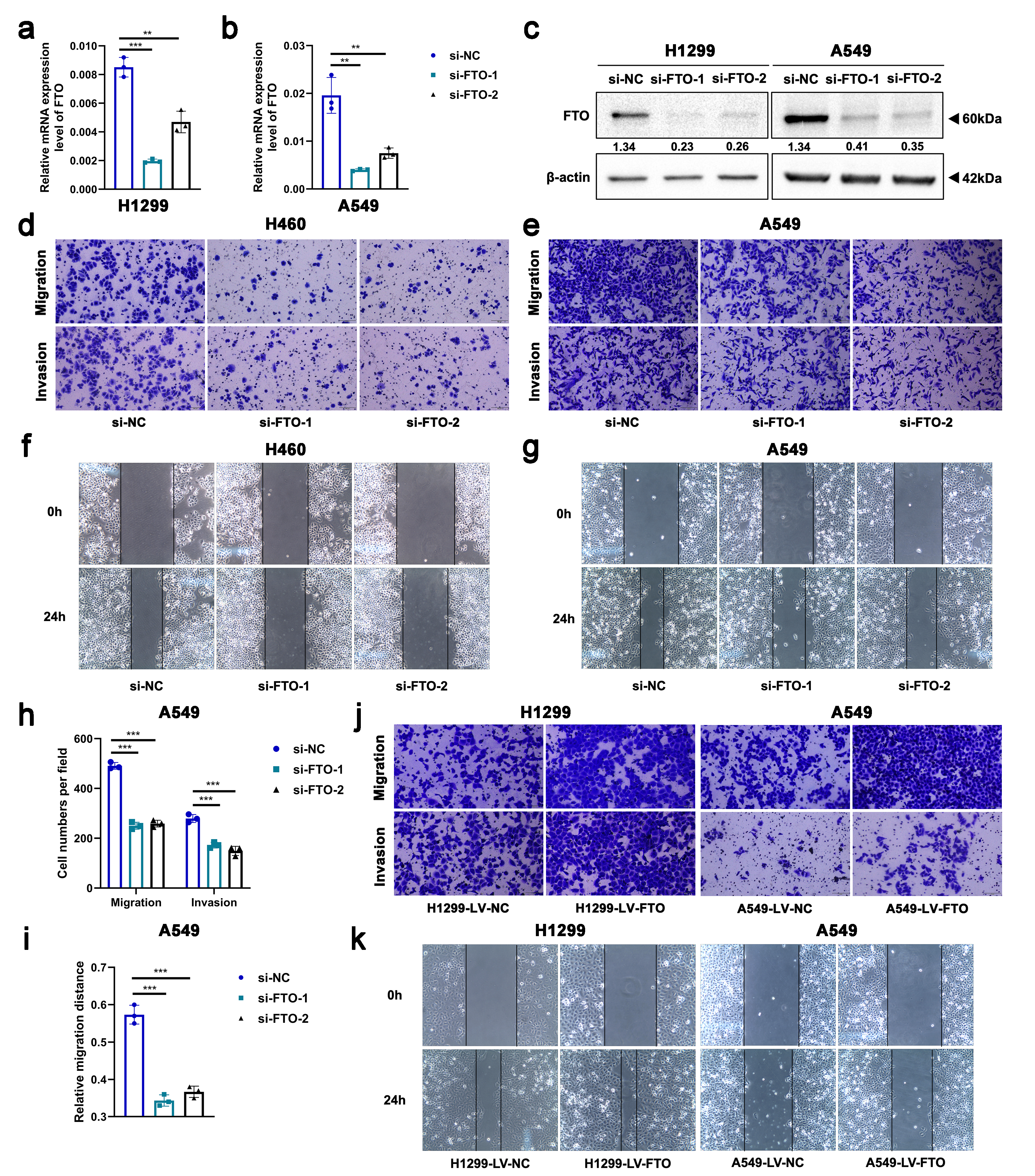

Supplement: Supplementary file 3 — Additional file 2: Figure S1. FTO promotes cell migration and invasion in NSCLC in vitro. a-b The relative mRNA expression levels of FTO in H1299 and A549 cells after transfection with siRNA. c The relative protein expression levels of FTO in H1299 and A549 cells after transfection with siRNA. d-e Representative images of the Transwell cell migration and invasion assays in H460 and A549 cells (si-FTO compared with si-NC). f-g Wound healing assays were performed to evaluate the role of FTO in H460 and A549 cells (si-FTO compared with si-NC). h-i Quantitative analysis of Transwell and wound healing assay data in FTO-knockdown A549 cells. j Representative images of the Transwell cell migration and invasion assays in H1299 and A549 cells (LV-FTO compared with LV-NC). k Wound healing assays were performed to evaluate the role of FTO in H1299 and A549 cells (LV-FTO compared with LV-NC). Data information: Data are shown as the mean ± SDs. In all relevant panels, *P < 0.05; **P < 0.01; ***P< 0.001. [file 12964_2023_1343_MOESM2_ESM.tif]

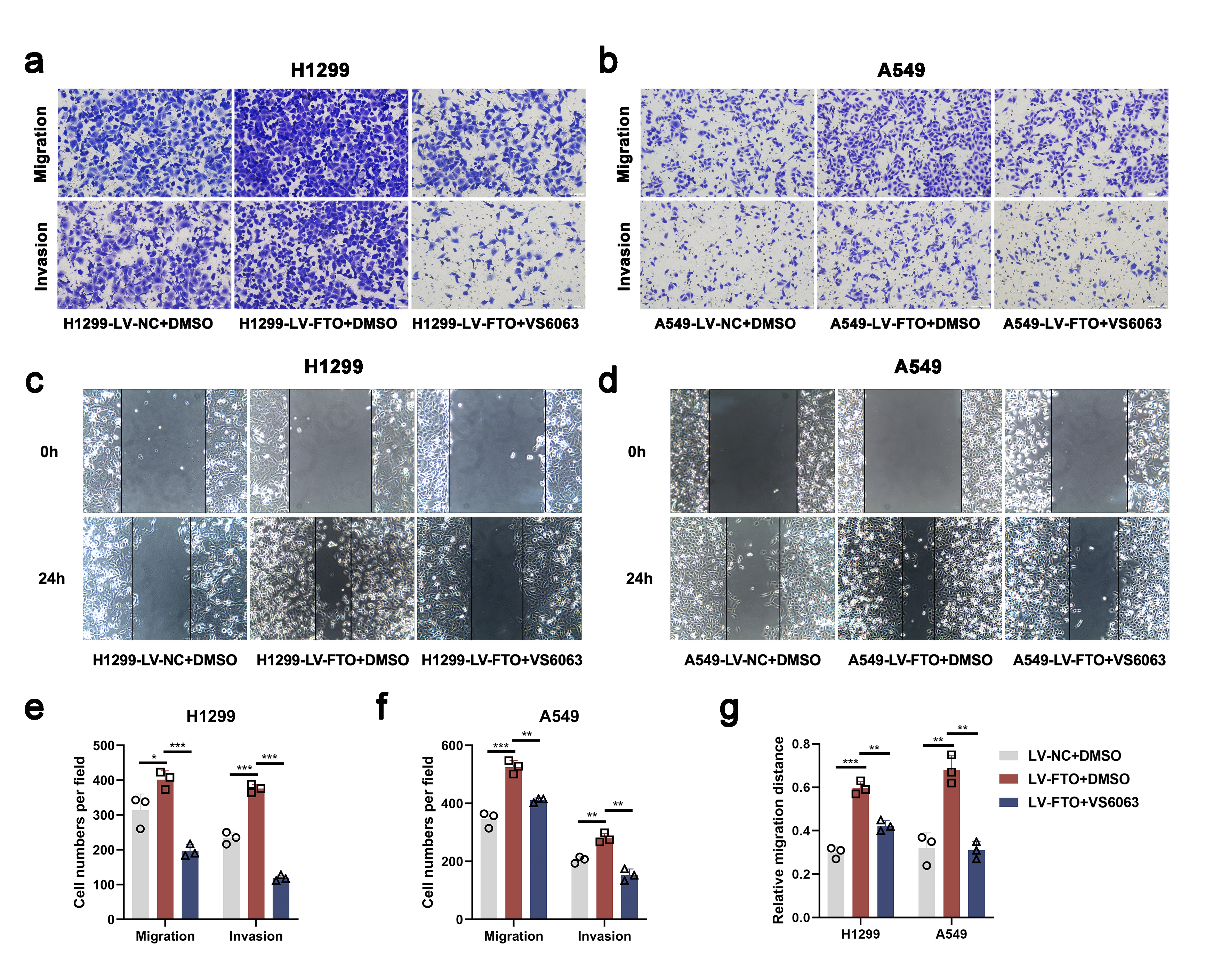

Supplement: Supplementary file 4 — Additional file 3: Figure S2. The FAK inhibitor defactinib (VS6063) inhibits the FTO-induced cell migration and invasion. a-b Representative images of the Transwell cell migration and invasion assays in H1299 and A549 cells. c-d Wound healing assays were performed to evaluate the role of VS6063 in H1299 and A549 cells. e-g Quantitative analysis of Transwell and wound healing assay data in H1299 and A549 cells. Data information: Data are shown as the mean ± SDs. In all relevant panels, *P < 0.05;**P < 0.01; ***P < 0.001. [file 12964_2023_1343_MOESM3_ESM.tif]

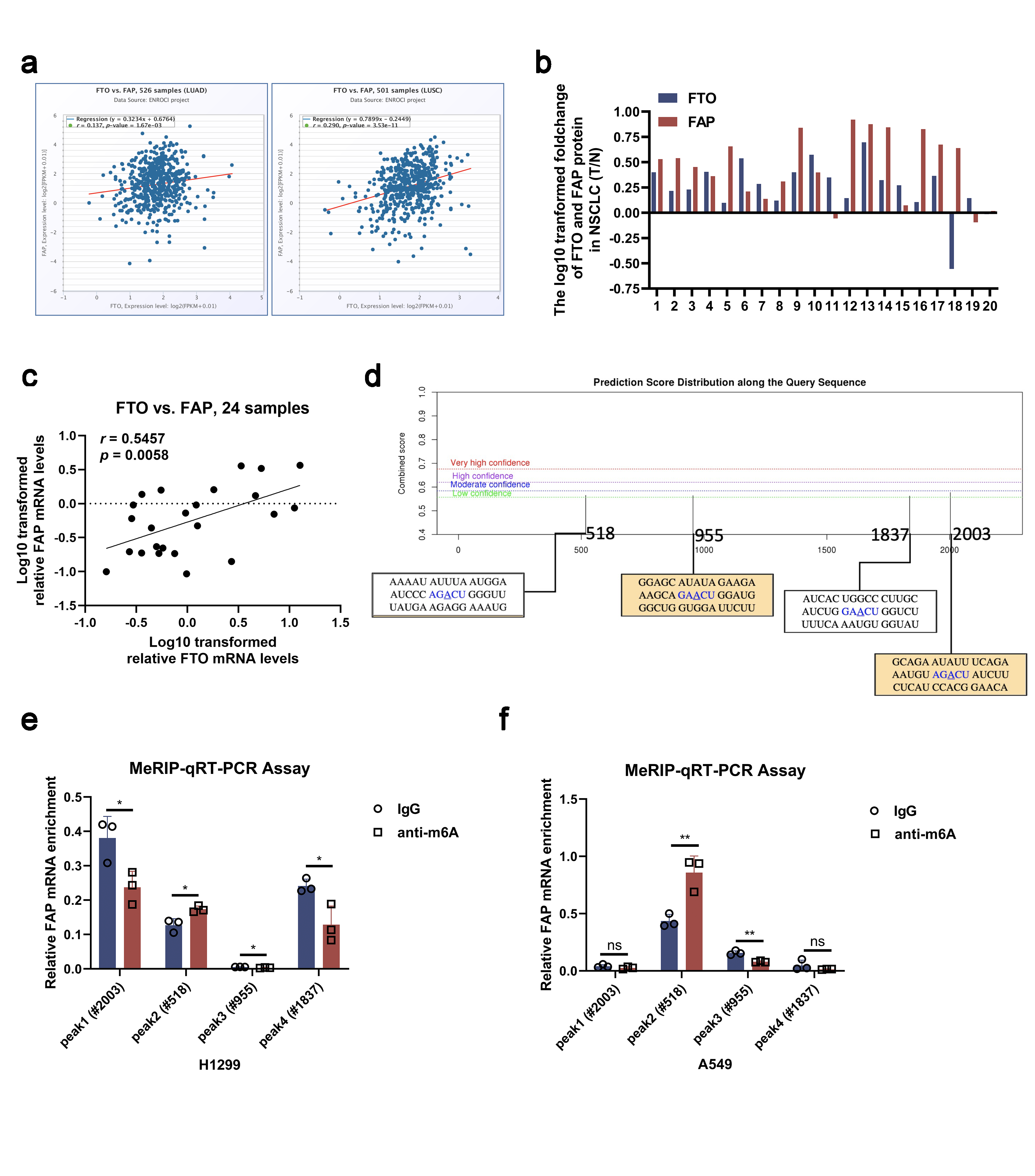

Supplement: Supplementary file 5 — Additional file 4: Figure S3. FTO modifies the m6A level of FAP. a Correlation analysis of the mRNA expression levels of FTO and FAP in NSCLC. Data were obtained from starBase database (https://starbase.sysu.edu.cn). b Relative quantification of FTO and FAP protein expression levels in 20 paired NSCLC tissues and adjacent tissues. The Y-axis shows the log10-transformed fold change in the T/N expression ratio. The X-axis shows the sample number. c Correlation analysis of the mRNA expression levels of FTO and FAP in NSCLC tumor samples based on qRT‒PCR. d M6A modification site prediction. Four m6A sites in FAP were predicted with SRAMP (http://www.cuilab.cn/sramp). e MeRIP qRT‒PCR assay, using an anti-m6A antibody or IgG to detect the binding to FAP in H1299 cells by 4 primers. IgG was used as the negative control. f MeRIP qRT‒PCR assay, using an anti-m6A antibody or IgG to detect the binding to FAP in A549 cells by 4 primers. IgG was used as the negative control. Data information: Data are shown as the mean ± SDs. In all relevant panels, *P < 0.05; **P < 0.01; ***P< 0.001. [file 12964_2023_1343_MOESM4_ESM.tif]

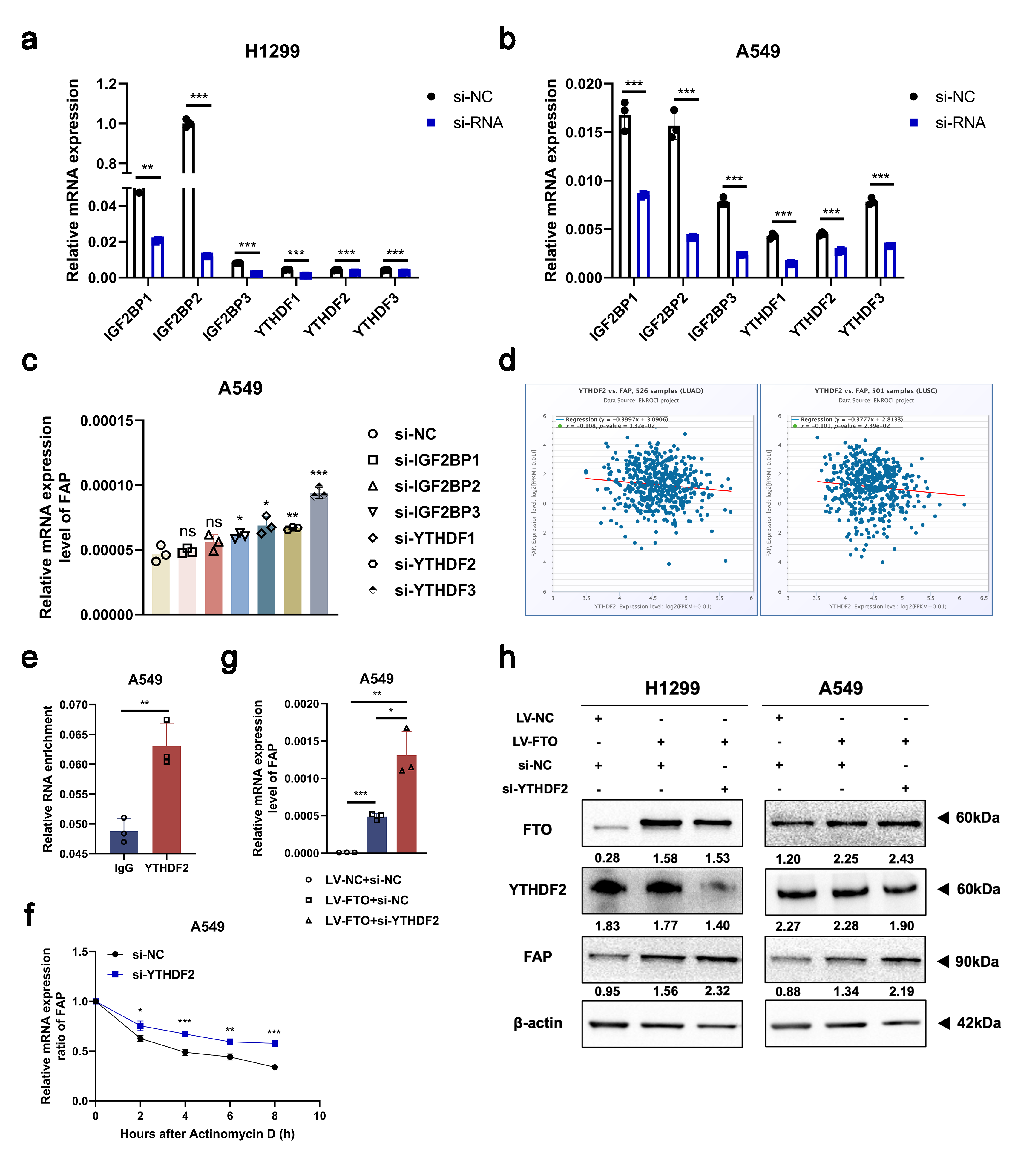

Supplement: Supplementary file 6 — Additional file 5: Figure S4. YTHDF2 is involved in FTO-mediated m6A demethylation modification in NSCLC. a-b The relative mRNA expression levels of the indicated genes in H1299 and A549 cells after transfection with the indicated constructs. c The mRNA expression levels of FAP were analyzed by qPCR in A549 cells transfected with the indicated constructs. d Correlation analysis of the mRNA expression levels of YTHDF2 and FAP in NSCLC. Data were obtained from the starBase database (https://starbase.sysu.edu.cn). e RIP assays, using an anti-YTHDF2 antibody or IgG to detect the binding to FAP in A549 cells. IgG was used as the negative control. f The relative mRNA expression ratio of FAP was analyzed by qPCR in actinomycin D-treated A549 cells at various time points (2, 4, 6, and 8 hours). g The relative mRNA expression levels of FAP in A549 cells transfected with the indicated constructs. h Western blot analysis verified the increased protein expression levels of FAP in FTO-overexpressing NSCLC cells after YTHDF2 knockdown. Data information: Data are shown as the mean ± SDs. In all relevant panels, *P< 0.05; **P < 0.01; ***P < 0.001. [file 12964_2023_1343_MOESM5_ESM.tif]

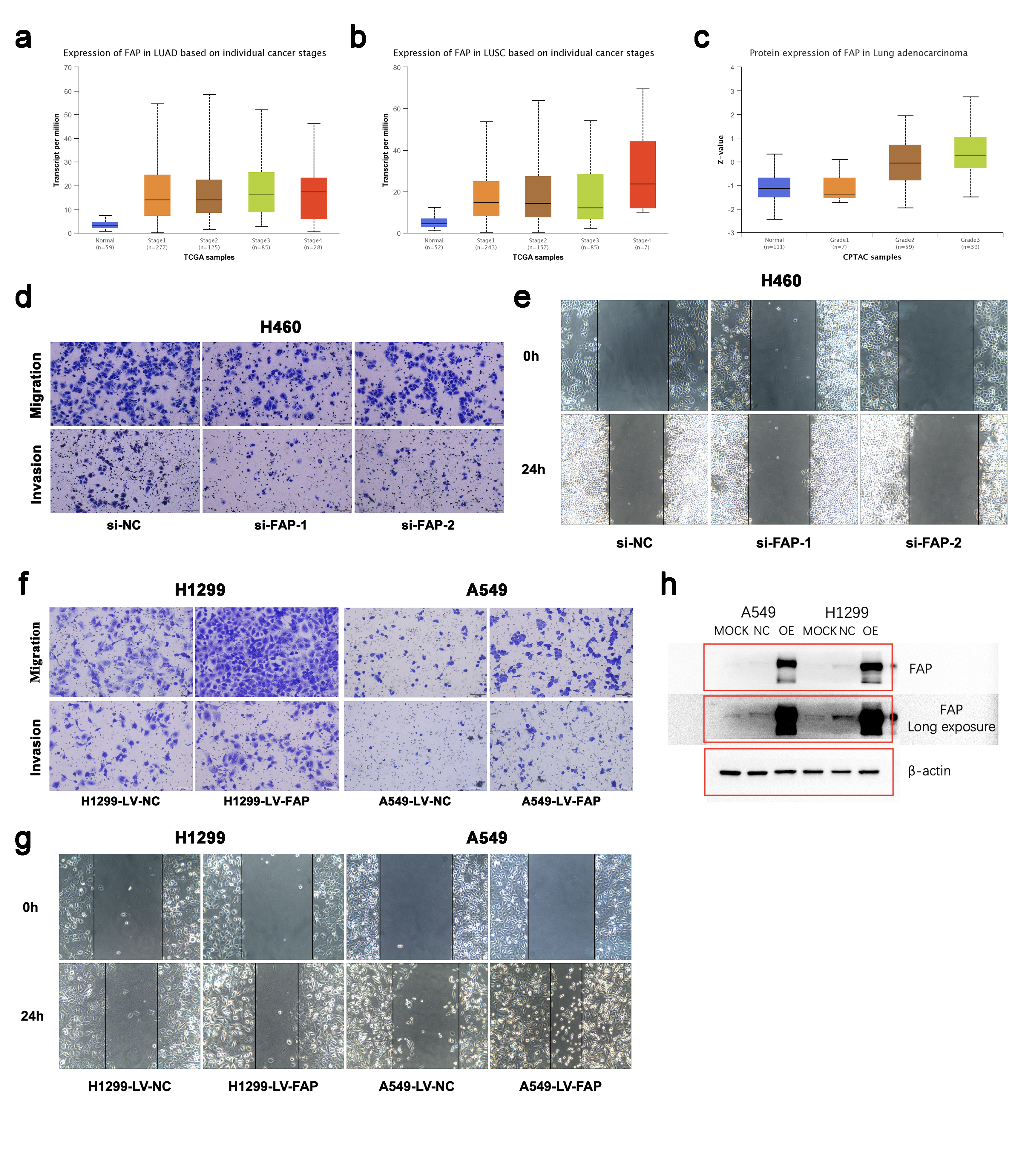

Supplement: Supplementary file 7 — Additional file 6: Figure S5. FAP promotes cell migration and invasion in NSCLC in vitro. a-b FAP mRNA expression levels were significantly upregulated in LUAD and LUSC, and the higher the clinical stage was, the higher the expression levels of FAP. Data were obtained from the online UALCAN database (http://ualcan.path.uab.edu). c The FAP protein expression levels were significantly upregulated in LUAD, the higher the clinical stage was, the higher the expression levels of FAP. Data were obtained from the online UALCAN database (http://ualcan.path.uab.edu). d Representative images of the Transwell cell migration and invasion assays in H460 cells (si-FAP compared with si-NC). e Wound healing assays were performed to evaluate the role of FAP in H460 cells (si-FAP compared with si-NC). f Representative images of the Transwell cell migration and invasion assays in H1299 and A549 cells (LV-FAP compared with LV-NC). g Wound healing assays were performed to evaluate the role of FAP in H1299 and A549 cells (LV-FAP compared with LV-NC). h Long-exposure images of FAP protein bands in Fig. 4. [file 12964_2023_1343_MOESM6_ESM.tif]

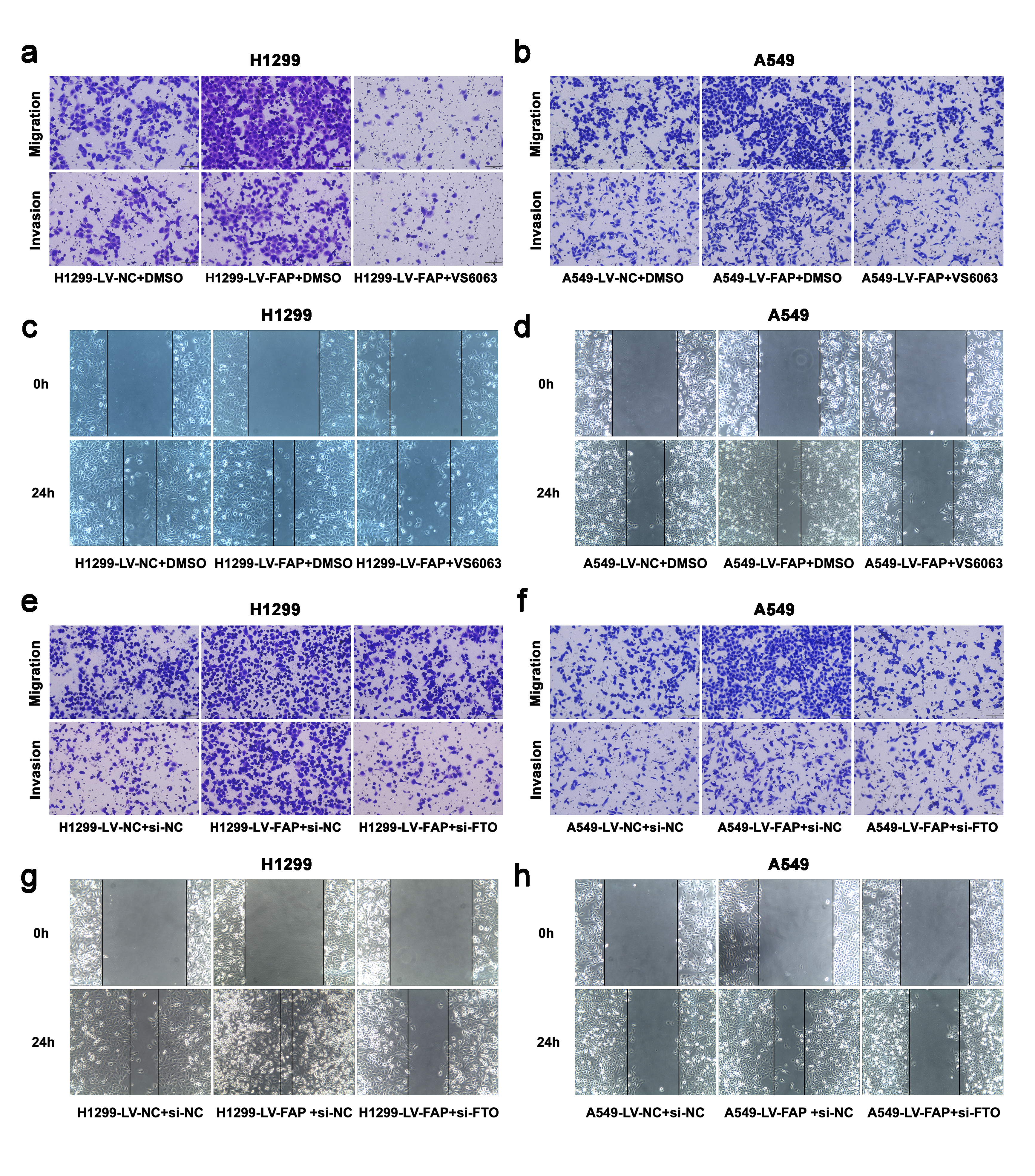

Supplement: Supplementary file 8 — Additional file 7: Figure S6. The FAK inhibitor defactinib (VS6063) inhibits the FAP-induced cell migration and invasion and rescue experiments. a-b Representative images of the Transwell cell migration and invasion assays in H1299 and A549 cells. c-d Wound healing assays were performed to evaluate the role of VS6063 in H1299 and A549 cells. e-f Representative images of the Transwell cell migration and invasion assays in H1299 and A549 cells. g-h Wound healing assays were performed in H1299 and A549 cells. [file 12964_2023_1343_MOESM7_ESM.tif]

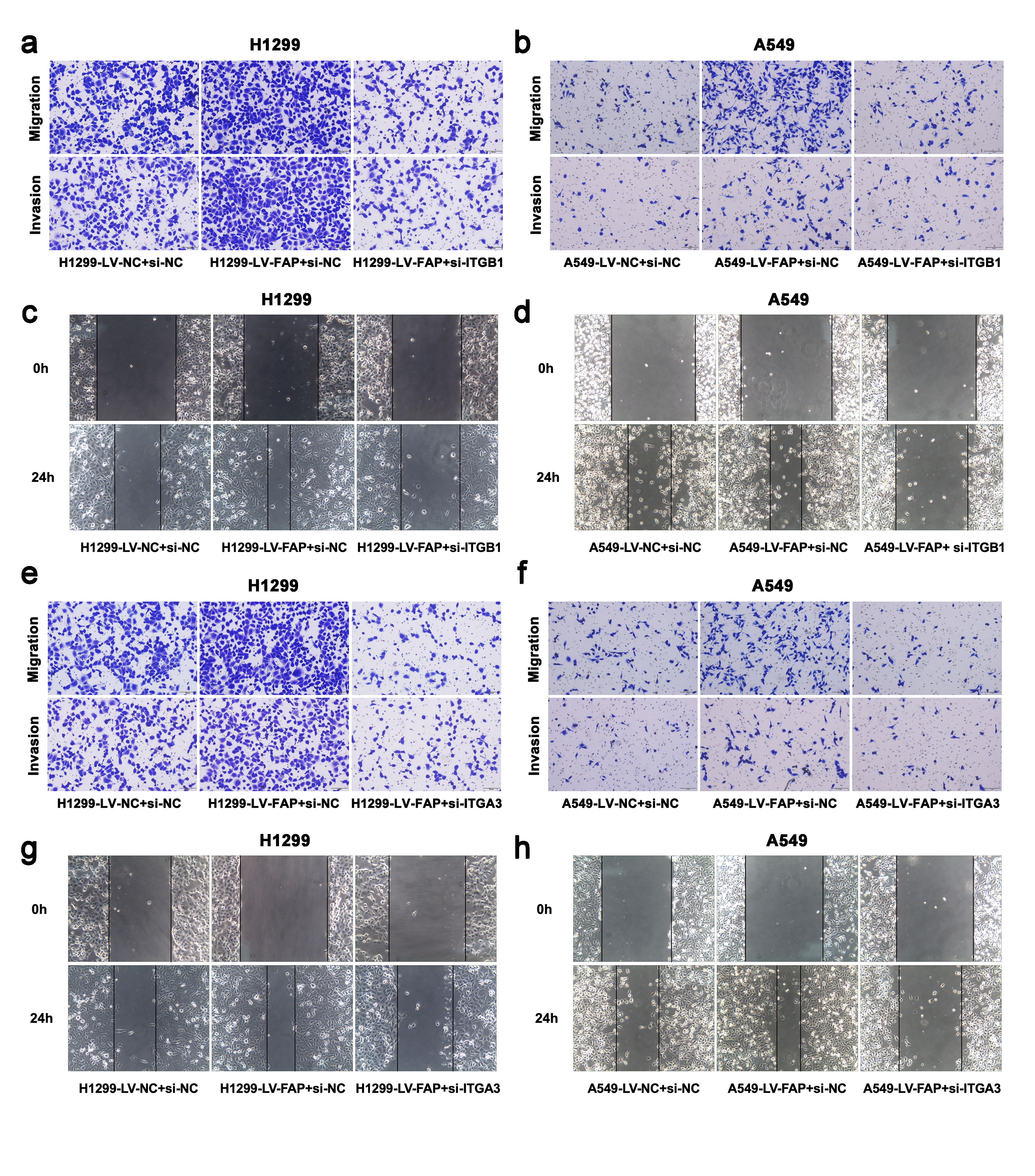

Supplement: Supplementary file 9 — Additional file 8: Figure S7. Integrin signaling is involved in FAP-induced FAK pathways activation. a-b, e-f Representative images of the Transwell cell migration and invasion assays in H1299 and A549 cells. c-d, g-h Wound healing assays were performed in H1299 and A549 cells. [file 12964_2023_1343_MOESM8_ESM.tif]
